# Identification and functional characterization of the *PP2C* gene family reveal their negative regulatory roles in stress adaptation in Solanaceae

**DOI:** 10.1186/s12870-026-09565-9

**Published:** 2026-08-06

**Authors:** Hong Chen, Yang Yang, Dehan Chen, Qian He, Chengjie Li, Binghui Zhang, Mingjian Chen, Yani Li, Gang Gu, Wenqing Li, Xiaofang Xie

**Affiliations:** 1 College of Life Sciences, Fujian Agriculture & Forestry University, Fuzhou, 350002 China; 2 Sanming Tobacco Company of Fujian Province, Sanming, 353000 China; 3 Institute of Tobacco Science, Fujian Provincial Tobacco Company, Fuzhou, 350003 China; 4 Tobacco-Rice Synergistic Development Industrial Technology Engineering Center, State Tobacco Monopoly Administration (China National Tobacco Corporation), Fuzhou, 350003 China; 5 Fujian Key Laboratory of Crop Breeding by Design, Fujian Agriculture & Forestry University, Fuzhou, 350002 China

**Keywords:** Protein phosphatase 2C, Stress response, Expression, Tobacco, Eggplant

## Abstract

**Background:**

The plant protein phosphatases 2C (PP2Cs) are key regulators of plant growth and development, hormone signaling, and responses to environmental stresses. In Solanaceae crops, which are frequently challenged by environmental stresses leading to yield losses, a comprehensive cross-species understanding of PP2C family evolution and function remains limited. This study aimed to systematically identify *PP2C* gene family members in tobacco and eggplant and to perform comparative genomic and functional analyses across representative Solanaceae species, thereby clarifying their evolutionary relationships and stress-responsive roles.

**Results:**

In this study, we identified 204 *NtPP2C* genes in tobacco (*Nicotiana tabacum* L.) and 89 *SmPP2C* genes in eggplant (*Solanum melongena* L.). The *PP2C* genes in both species were unevenly distributed across chromosomes, and segmental duplication appeared to be the major driving force underlying family expansion, with most homologous gene pairs subjected to purifying selection. Phylogenetic analysis classified these PP2C proteins into 12 subgroups (A–L). Members within different subgroups exhibited both conserved and divergent features in conserved motifs, exon-intron organization, and the composition of upstream promoter elements. Analyses of *cis*-acting elements, protein-protein interaction networks, and gene expression profiles indicated that *NtPP2C* and *SmPP2C* genes are broadly involved in light responses, developmental regulation, ABA signaling, drought, cold and heat stress responses, and pathogen infection-related processes. Subcellular localization analysis showed that NtPP2C91 and SmPP2C24, both belonging to the functionally important subgroup A, were localized to the nucleus. Further functional validation showed that CRISPR/Cas9-mediated knockout of *NtPP2C91* in tobacco and virus-induced gene silencing (VIGS) of *SmPP2C24* in eggplant significantly enhanced plant tolerance to low temperature and resistance to *Ralstonia solanacearum* (*Ras*) infection, while modulating the transcriptional dynamics of ABA signaling components and *PP2C* homologs.

**Conclusions:**

These findings provide valuable genetic resources and a theoretical basis for dissecting ABA-mediated stress-resistance regulatory networks in solanaceous crops and offer potential targets for molecular breeding aimed at improving stress tolerance.

**Supplementary Information:**

The online version contains supplementary material available at https://doi.org/10.1186/s12870-026-09565-9.

## Introduction

Plants are continuously challenged by both abiotic stresses, such as chemical, hydric, and thermal stressors, and biotic stresses, including pests, pathogens, and herbivory [1–4]. To cope with these stresses, they have evolved a range of adaptive strategies. A fundamental molecular mechanism that underlies plant adaptation is reversible protein phosphorylation [5]. This intricate process is precisely regulated by two key types of enzymes: protein kinases and protein phosphatases. Protein kinases act as “activators” by adding phosphate groups to specific proteins. In contrast, protein phosphatases serve as “de-activators” by removing these phosphate groups, allowing for a dynamic and reversible regulation of protein activity [6]. Among plant phosphatases, the protein phosphatases 2C (PP2Cs) represent the largest family, with important roles in plant signaling [7]. Notably, PP2C enzymes function as monomeric serine/threonine phosphatases, with catalytic activity dependent on Mg²⁺ or Mn²⁺ ions [8]. Plant PP2C family proteins possess a unique structure characterized by a conserved catalytic domain located at either the N-terminus or C-terminus, with the opposite end containing a non-conserved extension region of variable length [9]. These specialized structural features represent an important basis for the functional diversification of PP2C family proteins [10, 11]. For example, the N-terminal extension of subgroup B PP2C phosphatases contains a mitogen-activated protein kinase (MAPK) interaction motif that mediates MAPK binding and inactivation [11]. Similarly, the N-terminus of the *Arabidopsis* PP2C member DBP1 can bind promoters of defense-related genes, thereby contributing to transcriptional regulation associated with plant–virus interactions [12].

The *PP2C* gene family has been widely recognized as a key regulator of plant growth and development, as well as plant responses to biotic and abiotic stresses [11]. Accumulating evidence has demonstrated the diverse biological functions of *PP2C* genes in different plant species. For example, overexpression of the rice *PP2C* gene *OsBIPP2C2a* in transgenic tobacco enhances resistance to tobacco mosaic virus [13], whereas knockout of *NtPP2C171* in tobacco markedly improves drought tolerance [14]. In tomato, suppression of *SlPP2C1* significantly accelerates fruit ripening [15]. Given their essential roles, PP2C family members have been systematically identified in a wide range of plant species, including 80 *PP2Cs* in *Arabidopsis* [16], 78 in rice [16], 181 in *Gossypium hirsutum* [17], 56 in cucumber [18], 257 in wheat [19], and 131 in *Brassica rapa* [20]. Based on sequence similarity and phylogenetic relationships, plant PP2C proteins are generally classified into 12 subgroups, designated A to L, along with an ungrouped class [7, 19]. These subgroups differ not only in the number of PP2C members they contain but also in their functional characteristics [8]. For instance, subgroup A *PP2Cs* are primarily involved in abscisic acid signaling [21], whereas subgroup B members participate in the regulation of MAPK pathways [22, 23].

Solanaceous crops are of considerable economic importance, yet their production is frequently constrained by diverse biotic and abiotic stresses, leading to substantial losses in yield and quality. The *PP2C* gene family is widely involved in plant stress responses and represents a promising source of target genes for improving stress tolerance [24–26]. Genome-wide analyses of *PP2C* genes have been reported in several solanaceous species, including tomato, potato, and tobacco, providing an important foundation for understanding this gene family. However, most existing studies have focused on individual species, and a systematic cross-species comparison of *PP2C* genes across multiple solanaceous crops under a unified analytical framework remains limited. In this study, we systematically identified *PP2C* gene family members in tobacco and eggplant and integrated these results with available data from tomato and potato to establish a comparative phylogenetic and syntenic framework for four solanaceous species. We further focused on *PP2C* genes belonging to subgroup A and compared the stress-responsive patterns of candidate genes in tobacco and eggplant under abscisic acid treatment, low-temperature stress, and *Ralstonia solanacearum* (*Ras*) challenge, followed by functional validation. The findings not only expand the genomic resources for the eggplant PP2C family but also offer cross-species evidence that illuminates the evolutionary relationships and stress-response roles of PP2C genes across the Solanaceae.

## Materials and methods

### Genome identification of *PP2C* gene family members in tobacco and eggplant

Protein sequences of tobacco (NtaSR1) were downloaded from Nicomics (http://lifenglab.hzau.edu.cn/Nicomics/index.php) [27], while eggplant protein sequences (Eggplant-HQ) were obtained from the Eggplant Genome Database (http://47.92.172.28:12068/Eggplant/home/index). First, AtPP2C protein sequences retrieved from the Arabidopsis Information Resource (TAIR, https://www.arabidopsis.org/) were used as queries for local BLASTP searches. Candidate PP2C proteins were initially screened using thresholds of sequence similarity ≥ 30% and E-value ≤ 1e^− 5^. In parallel, a complementary search was performed using the Hidden Markov Model (HMM) profile of the PP2C domain (PF00481) obtained from the Pfam database (http://pfam.xfam.org/). Candidate sequences identified by the two methods were integrated. For genes with multiple transcript IDs, only the longest transcript was retained. Incomplete gene sequences lacking start or stop codons and sequences containing aberrant mutations were excluded. The remaining candidates were then verified for conserved domains using the NCBI Conserved Domain Database (CDD, https://www.ncbi.nlm.nih.gov/cdd) [28]. Subsequently, the physicochemical properties of the identified PP2C proteins in tobacco and eggplant were analyzed using the ExPASy online tool (https://www.expasy.org) [29], and their subcellular localization was predicted with the Cell–PLoc 2.0 (http://www.csbio.sjtu.edu.cn/bioinf/Cell-PLoc-2/) [30].

### Chromosome location and collinearity analysis

Chromosomal localization information of tobacco (NtaSR1) was obtained from the Nicomics database (http://lifenglab.hzau.edu.cn/Nicomics/index.php), while that of eggplant (Eggplant-HQ) was retrieved from the Eggplant Genome Database (http://47.92.172.28:12068/Eggplant/home/index). Chromosomal localization data of tomato (ITAG4.0) and potato (SolTub_3.0) were acquired from the Phytozome database (https://phytozome.jgi.doe.gov/pz/portal.html). Based on these datasets, intra-genomic and inter-genomic collinearity analyses of the PP2C gene family were performed using MCScanX software (default parameters) [31]. The analysis results were visualized and the non-synonymous (*Ka*) and synonymous (*Ks*) substitution rates were calculated using TBtools-II software (version 2.2.10) [32]. In addition, the divergence time of homologous gene pairs was estimated using the formula T = *Ks / 2λ* (assuming a clock-like rate (*λ*) of 6.96 × 10⁻⁹ synonymous substitutions per site per year) [33, 34].

### Phylogenetic analysis

To elucidate the evolutionary relationships of the PP2C gene family in Solanaceae species, multiple sequence alignment of PP2C protein sequences from tobacco, eggplant, tomato, and potato was performed using MUSCLE [35–37]. The resulting alignment was trimmed using trimAl to remove poorly aligned regions [38]. A phylogenetic tree was subsequently constructed using the maximum likelihood (ML) method implemented in IQ-TREE [39], with 1000 bootstrap replicates and all other parameters set to default. The resulting phylogenetic tree was annotated and visually refined using the iTOL web tool (https://itol.embl.de/) [40].

### Analysis of protein conserved motifs and gene structure

Conserved motifs in the NtPP2C and SmPP2C proteins were analyzed using the MEME online tool (http://meme-suite.org/tools/meme) [41], with parameters set to identify 20 motifs and motif lengths ranging from 6 to 50 amino acids. Motif annotation was performed using the Pfam database. Additionally, the exon-intron structures of the *NtPP2C* and *SmPP2C* genes were examined using TBtools-II [32].

### Analysis of *cis*-acting elements in *PP2C* gene promoters

The upstream 2000 bp sequences from the start codons of *NtPP2C* and *SmPP2C* genes were extracted using the GXF Sequences Extract tool in TBtools-II for promoter analysis [32]. These sequences were then submitted to the PlantCARE online website (http://bioinformatics.psb.ugent.be/webtools/plantcare/html/) [42] for predicting *cis*-acting elements.

### Protein interaction network of NtPP2Cs and SmPP2Cs

Based on the homology between AtPP2C proteins and their counterparts in tobacco and eggplant, protein-protein interaction (PPI) networks among PP2C family members were predicted using the STRING database (https://string-db.org/). The resulting networks were subsequently visualized and analyzed with Cytoscape 3.10.3.

### Transcriptome analysis of *NtPP2C* and *SmPP2C* genes

Transcriptome profiling has been widely used to identify candidate regulatory genes in crops [43–46]. To investigate the tissue-specific expression and stress responses of *PP2C* genes, the RNA-seq data for the *NtPP2C* genes in different tissues (GSE233199) [47] and under drought conditions (GSE214048) [48] were retrieved from NCBI (https://www.ncbi.nlm.nih.gov/geo/). Similarly, the RNA-seq data for the *SmPP2C* genes in various tissues (PRJNA839598) and under high temperature conditions (PRJNA1044156) [49] were also obtained. Expression heatmaps for *NtPP2C* and *SmPP2C* genes were generated using the HeatMap function in TBtools-II [32]. Gene expression trends were further visualized using the OmicShare online tool (https://www.omicshare.com/tools/) [50].

### Subcellular localization

The complete coding sequences of *NtPP2C91* and *SmPP2C24*, excluding the stop codons, were cloned into the pSuper1300-GFP vector. The resulting recombinant constructs were introduced into *Agrobacterium* strain GV3101. Healthy *Nicotiana tabacum* cv. K326 plants were used as experimental material. A mixture of the recombinant construct suspension and the nuclear marker NLS-mCherry was infiltrated into the abaxial side of K326 leaves using a sterile syringe, avoiding the main veins. Following infiltration, the plants were subjected to dark treatment for 36 h. The infiltrated leaves were subsequently excised into appropriately sized segments, rinsed with sterile water, and mounted on slides. Fluorescence signals in the leaf tissue were examined using a laser scanning confocal microscope (STELLARISS; Leica).

### Gene knockout and silencing material construction

CRISPR/Cas9-mediated genome editing was used to generate *NtPP2C91* knockout plants. A gRNA target sequence (TGGGATGGACCAAGGGTTCTTGG) was designed following the manufacturer’s instructions for the CRISPR/Cas9 genome-editing kit (Catalog No. VK005-1, Vazyme Biotech Co., Ltd., Beijing, China) and cloned into the pEGAtCas9PcUBI-H vector. The recombinant plasmid was propagated in *Escherichia coli* DH5α, verified by Sanger sequencing, and introduced into *Agrobacterium* GV3101 for transformation of *Nicotiana tabacum* cv. K326. Genomic DNA was extracted from T0 transgenic plants using a Plant Genomic DNA Extraction Kit (Catalog No. BL1042A, Biosharp, China). The target region was amplified by PCR, and product specificity was confirmed by agarose gel electrophoresis before Sanger sequencing (Sangon Biotech, Shanghai, China). Mutation types were determined based on sequencing chromatograms to confirm successful disruption of *NtPP2C91*. T0 plants were self-pollinated to obtain T1 progeny, which were further validated by PCR and sequencing for subsequent experiments.

To assess the function of *SmPP2C24*, a tobacco rattle virus (TRV)-based virus-induced gene silencing (VIGS) assay was performed in the eggplant cultivar Yanzhiqie. The optimal silencing fragment was selected using the SGN VIGS tool (https://vigs.solgenomics.net/). The target fragment with adapter sequences was PCR-amplified, gel-purified, and ligated into the pTRV2 vector digested with BamHI and EcoRI to construct pTRV2-*SmPP2C24*. The construct was transformed into *E. coli* DH5α, and positive colonies were screened for plasmid preparation. Recombinant pTRV2-*SmPP2C24*, empty pTRV2, and the helper plasmid pTRV1 were introduced into *Agrobacterium* GV3101. VIGS plants were generated by *Agrobacterium*-mediated infiltration, with inoculation parameters following previously reported procedures [51, 52]. To assess systemic efficiency, a pTRV2 vector carrying the *GFP* gene sequence was included. In this system, robust GFP fluorescence detected in the leaves was interpreted as conclusive evidence of a successful viral infection (Figure S1). Primer sequences are listed in Table S1.

### Plant material cultivation and stress treatments

Tobacco (*Nicotiana tabacum* L.) cultivar K326 and eggplant (*Solanum melongena* L.) cultivar Yanzhiqie (YZQ; accession ID: V06B0149) were used in this study and were grown in a greenhouse at Fujian Agriculture and Forestry University. Greenhouse conditions were maintained at a light intensity of 400 µmol m⁻² s⁻¹, 25 °C, a photoperiod of 16 h of light and 8 h of darkness, and 70% relative humidity. After 4 weeks of growth, uniform and healthy seedlings were selected for subsequent experiments.

Plants of tobacco wild-type K326, eggplant wild-type YZQ, the successful *NtPP2C91* knockout T1 line (designated as *ntpp2c91)*, the empty vector control (pTRV2), and the successful VIGS silencing lines (pTRV2-*SmPP2C24*) were grown in pots and subjected to abscisic acid (ABA) treatment, cold stress, or biotic stress. For ABA treatment, plants were sprayed with 0.1 mM ABA solution and sampled after 12 h. For cold stress, plants were transferred to a 5℃ growth chamber for 12 h, while the other conditions were kept unchanged. For biotic stress, plants were inoculated with a highly efficient strain of *Ras* strain preserved by our laboratory [53] and then plants were cultured in a greenhouse with high-humidity and high-temperature (30℃, approximately 80% humidity, 12 h/d light).

All experiments were performed with three biological replicates, with five plants per replicate. Collected samples were wrapped in aluminum foil, rapidly frozen in liquid nitrogen, and stored at − 80℃ for subsequent RNA extraction.

### Quantitative real-time PCR analysis

Total RNA was extracted using the *SteadyPure* RNA Extraction Kit (Catalog No. AG21024, Accurate Biology, Hunan), and cDNA was synthesized with the Evo M-MLV RT Mix Kit (Catalog No. AG11728). Gene expression was quantified by quantitative reverse-transcription PCR (qRT–PCR) using SYBR Green Pro Taq HS Premixed qPCR Kit (Catalog No. AG11718), and relative expression levels were calculated with the 2^−ΔΔCt^ method [54]. Three technical replicates were performed for each sample. The actin genes from tobacco and eggplant were used as internal reference genes (Table S1).

## Results

### Characterization of *PP2C* genes in tobacco and eggplant genomes

Through similarity searches and conserved-domain analyses, 293 *PP2C* gene family members were identified, including 204 in tobacco and 89 in eggplant. These genes were sequentially named *NtPP2C01*–*NtPP2C204* and *SmPP2C01*–*SmPP2C89*, respectively (Table S2). In tobacco, the *NtPP2C* genes showed substantial structural variation, with exon numbers ranging from 1 to 20, the highest number being observed in *NtPP2C16*. The encoded proteins also varied markedly in length, from 175 amino acids (NtPP2C200) to 1083 amino acids (NtPP2C78 and NtPP2C87). Their molecular weights ranged from 19.84 kDa (*NtPP2C200*) to 121.62 kDa (*NtPP2C16*), and their theoretical isoelectric points ranged from 4.14 (NtPP2C23) to 9.43 (NtPP2C14). Similarly, the *SmPP2C* genes in eggplant exhibited extensive structural diversity, with exon numbers ranging from 1 to 21, with *SmPP2C73* containing the highest number of exons. The corresponding proteins ranged from 175 amino acids (SmPP2C15) to 1080 amino acids (SmPP2C73) in length, with molecular weights varying from 19.44 kDa (*SmPP2C15*) to 122.64 kDa (*SmPP2C73*). Their theoretical isoelectric points ranged from 3.87 (SmPP2C04) to 9.56 (SmPP2C74). These results indicate considerable variation in the structural and physicochemical properties of PP2C proteins in both tobacco and eggplant. Subcellular localization prediction showed that most NtPP2C and SmPP2C proteins were localized to intracellular compartments, including the nucleus, cytoplasm, chloroplasts, mitochondria, and peroxisomes, whereas only a small number were predicted to localize to the plasma membrane or cell wall. Among these predicted locations, the nucleus was the most frequent. This distribution underscores the organelle-specific roles of NtPP2C and SmPP2C proteins in cellular processes.

### Chromosome localization and collinearity analysis of *NtPP2C* and *SmPP2C* genes

**Fig. 1:**
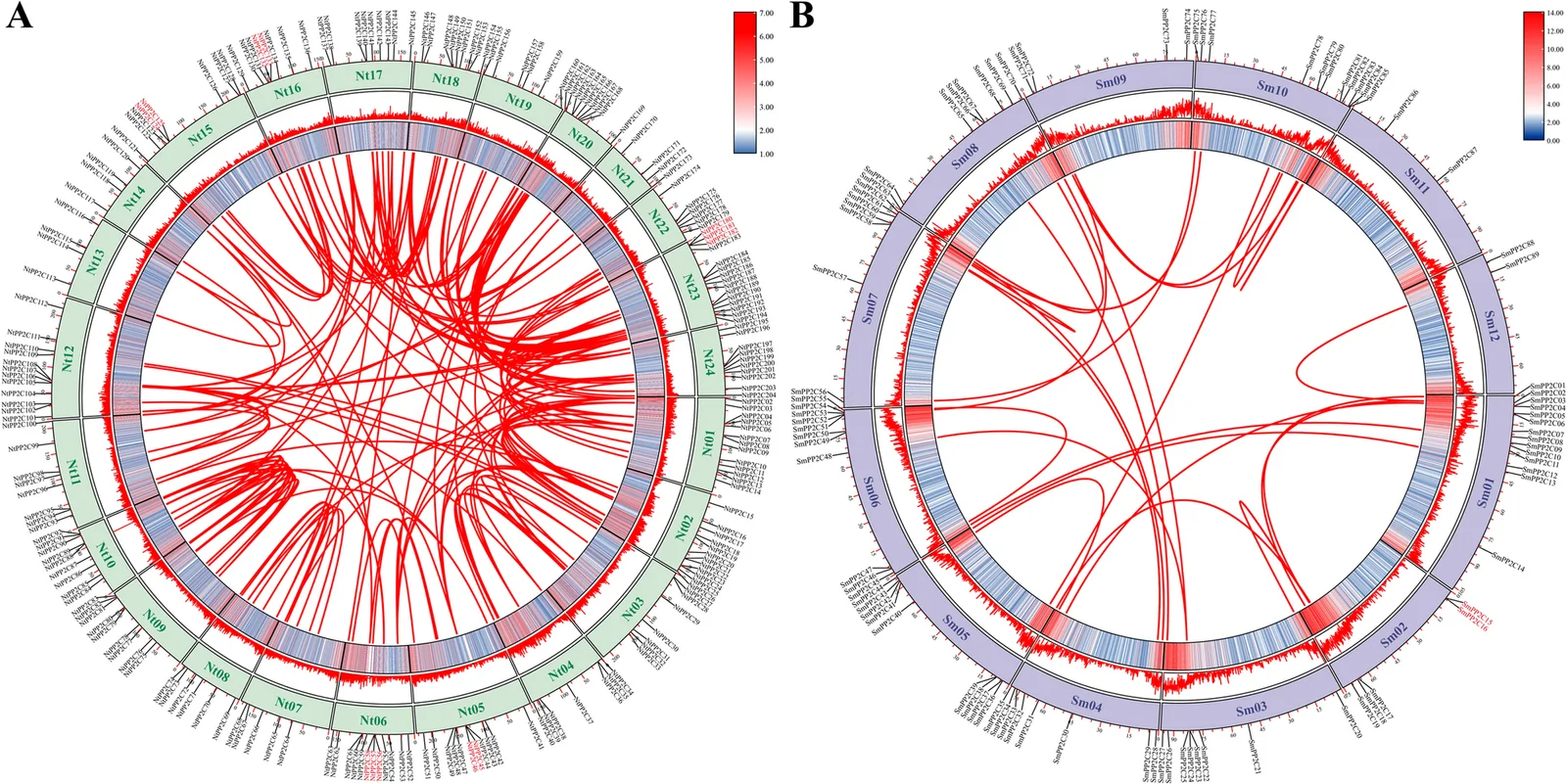
Analysis of the chromosomal distribution and duplication events of the *PP2C* gene family in tobacco and eggplant. **A** Chromosomal locations of *NtPP2C* genes in tobacco. **B** Chromosomal locations of *SmPP2C* genes in eggplant. Tandem duplicated genes are marked with red gene names, while segmental duplicated genes are connected by red lines

Chromosomal localization analysis showed that *PP2C* genes are unevenly distributed across the tobacco and eggplant genomes. In tobacco, 203 of the 204 identified *NtPP2C* genes were mapped to 24 chromosomes, whereas one gene was located on an unassigned scaffold (Fig. 1A). The number of *NtPP2C* genes varied considerably among chromosomes, with chromosome 23 carrying the largest number of genes (15), followed by chromosome 1 with 13 genes. By contrast, chromosomes 13, 14, and 21 each contained only four *NtPP2C* genes. A similar uneven distribution pattern was observed in eggplant, where 89 *SmPP2C* genes were distributed across 12 chromosomes (Fig. 1B). Chromosome 1 contained the highest number of *SmPP2C* genes (14), followed by chromosomes 6 and 10, each harboring 11 genes. The fewest *SmPP2C* genes were found on chromosomes 11 and 12, with only two genes on each chromosome. To further investigate the amplification mechanisms of the *PP2C* gene family, segmental and tandem duplication events were analyzed in the tobacco and eggplant genomes.

MCScanX identified a total of 170 duplicated gene pairs in tobacco, of which 161 were classified as segmental duplications (94.7%), while in eggplant, 25 of the 26 duplicated gene pairs were segmental duplicated (96.2%). This high proportion suggests that segmental duplication is the predominant mode of duplication detected within the PP2C families of both species. (Table S3). Additionally, *Ka*, *Ks*, and *Ka/Ks* for these 196 homologous gene pairs were calculated. In general, the value of *Ka/Ks* < 1 indicates purifying (negative) selection, *Ka/Ks* = 1 indicates neutral selection, and *Ka/Ks* > 1 indicates positive selection [55]. The results indicated that, except for the gene pair *SmPP2C15-SmPP2C16*, the *Ka/Ks* values of all other gene pairs were less than 1, indicating that these genes have primarily undergone purifying selection during the evolutionary process (Table S4). It should be noted, however, that because MCScanX-based syntenic blocks may include genes originating from whole-genome duplication events, we are unable to definitively distinguish between true segmental duplications and remnants of WGD based solely on the current dataset.

### Protein conserved motifs and gene structure analysis of NtPP2Cs and SmPP2Cs

To investigate the evolutionary relationship between *NtPP2C* and *SmPP2C* genes, a phylogenetic tree was constructed. The *NtPP2C* and *SmPP2C* genes were divided into 12 subgroups (A–L; Figure S2A). Due to the crucial role of conserved motifs and gene structure in the evolution of gene families, the conserved motifs and exon-intron structures of *PP2C* genes in tobacco and eggplant were further analyzed. A total of 20 conserved motifs were identified in 204 *NtPP2C* genes and 89 *SmPP2C* genes (Table S5). As shown in Figure S2B, motifs 1, 2, 3, 6, and 7 were present in nearly all *NtPP2C* and *SmPP2C* proteins, while motif 4 was absent only in the K subgroup, suggesting that these motifs play a critical role in the functional activity of *PP2C* genes. Within each subgroup, gene members exhibited similar motif compositions and arrangements, whereas differences were observed between subgroups. For instance, most genes in the H subgroup contained motifs 1, 2, 3, 4, 5, 6, 7, 11, 12, 13, and 18, while genes in the D subgroup primarily contained motifs 1, 2, 3, 4, 6, 7, 8, 9, 10, 11, 12, 13, and 15. Furthermore, some motifs were specific to certain subgroups. For example, motif 15 was found only in the D and L subgroups, motif 17 was exclusive to the C subgroup, and motif 19 was present only in the A and C subgroups. These findings suggest that the unique functions of genes within different subgroups may be determined by their distinct motif compositions. Further analysis of these 20 motifs using the Batch CD-search tool revealed that motifs 1, 2, 3, 4, and 5 were annotated as catalytic domains of the PP2C family [56], with motif 1 also identified as a component of the PTC1 domain, which is associated with plant growth and development [57].

Exon-intron structure analysis of *NtPP2C* and *SmPP2C* genes revealed that among the 293 genes examined, *SmPP2C73* had the highest number of exons, with a total of 21 (Figure S2C). The most common structure was the four-exon structure, found in 83 genes, accounting for 28.3% of the total. These results indicate significant differences in exon number and gene sequence length among gene family members. However, genes within the same subgroup exhibited similar structural characteristics, including exon number and exon length. For example, compared to other subgroups, members of the C subgroup generally had longer exon lengths, while members of the I subgroup had more exons. Overall, *PP2C* genes in both tobacco and eggplant exhibit a combination of evolutionary conservation and diversity.

### *Cis*-acting elements analysis of *NtPP2C* and *SmPP2C* genes

Transcriptional regulation of genes is mediated by the interaction of transcription factors with *cis*-acting elements in the upstream promoter region. Therefore, the study of *cis*-acting elements in the promoter regions of *PP2C* genes in tobacco and eggplant can provide deeper insights into the potential functions of these genes in plant development and under various stress conditions. To gain a comprehensive understanding, the upstream 2000 bp promoter regions of *NtPP2C* and *SmPP2C* genes were analyzed. A total of 40 *cis*-acting elements were identified, which can be classified into three categories: plant growth and development elements, abiotic/biotic stress responsive elements, and phytohormone responsive elements (Figure S3, Table S6). Growth and development elements, such as Box 4, G-box, GT1-motif, L-box, TCT-motif, GA-motif, TCCC-motif, chs-CMA1a, and GATA-motif, were found to be widely distributed in the promoter regions of *NtPP2Cs* and *SmPP2Cs*, with most of them associated with light response. This suggests that *NtPP2Cs* and *SmPP2Cs* play key roles in photoperiod and circadian rhythms. The promoter regions also contain a large number of stress responsive elements, including LTR related to low-temperature response, W-box associated with pathogen induction, as well as MBS and DRE core elements related to drought response. Additionally, phytohormone‑responsive elements were detected in the promoters of *NtPP2C* and *SmPP2C* genes, among which the abscisic acid response element (ABRE) was the most prevalent. This suggests a potential role for these genes in ABA‑mediated signaling pathways.

### Protein interaction network of NtPP2Cs and SmPP2Cs

**Fig. 2:**
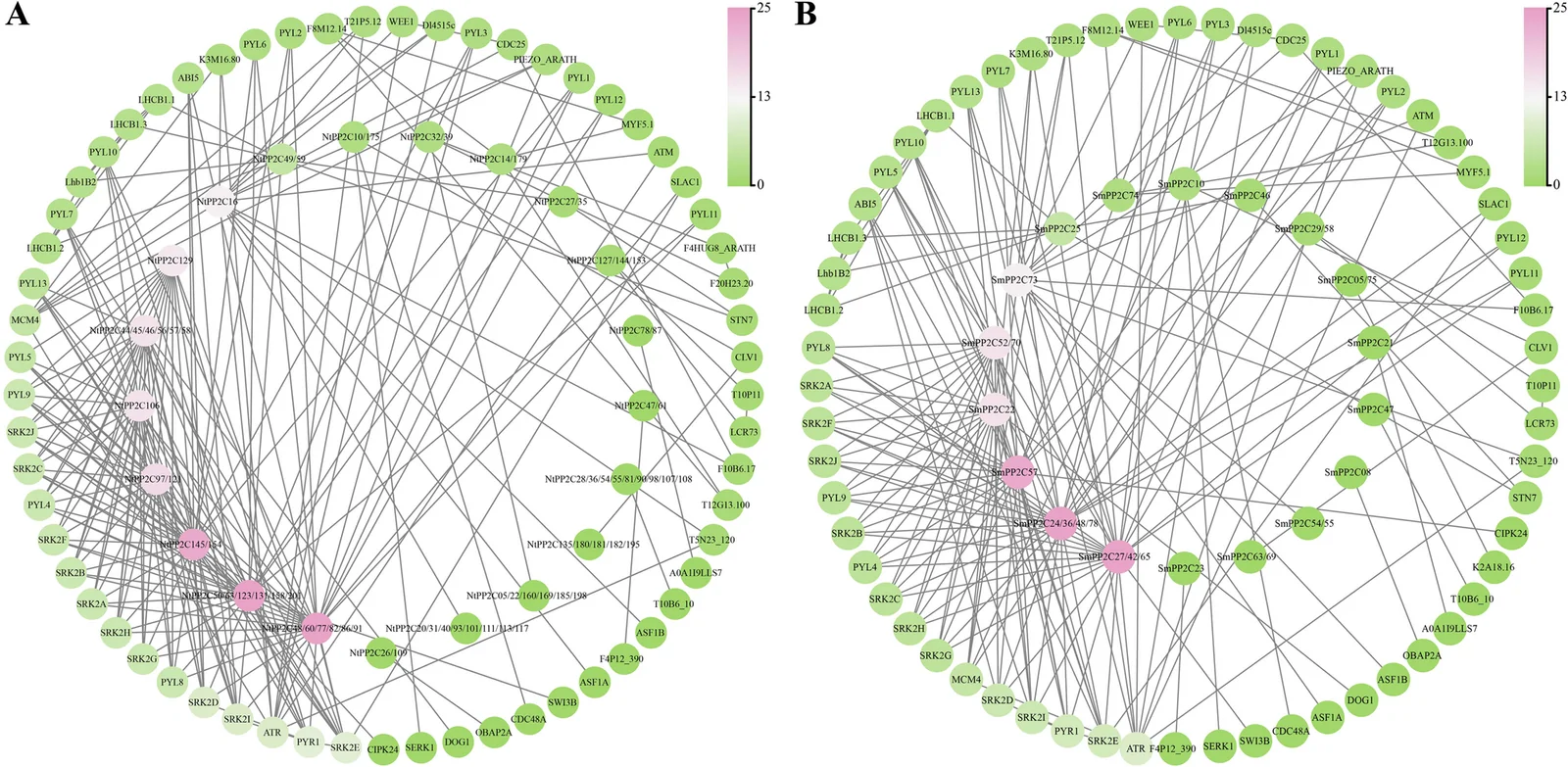
Functional interaction networks of (**A**) NtPP2Cs and (**B**) SmPP2Cs according to orthologues in *Arabidopsis*. Network nodes represent proteins, and gray lines represent protein-protein associations. The node color represents the number of proteins that interact with each other

To explore potential regulatory mechanisms, protein‑protein interaction (PPI) networks of PP2C proteins in tobacco and eggplant were predicted based on homology with Arabidopsis counterparts. The network analysis indicated that PP2C proteins within the same species exhibited mutual interactions with one another and also interacted with multiple proteins implicated in diverse functional processes (Fig. 2). Notably, NtPP2C and SmPP2C proteins were predicted to interact with photosystem II-related proteins (LHCB1.1, LHCB1.2, and LHCB1.3) [58], development-related proteins (ASF1A, ASF1B, and WEE1) [59, 60], stress response-related proteins (DOG1 and PYL3) [61, 62], ABA receptor family proteins (PYRABACTIN RESISTANCE 1 [PYR1]/PYR1-LIKE [PYLs]), and subclass III SnRK2 kinases (SRK2s) [63], among others. These interactions suggest that PP2C proteins in tobacco and eggplant are involved in the modulation of photosynthesis, growth and development, stress tolerance, and hormone signaling pathways. However, because this network is not based on experimentally validated physical interactions in tobacco or eggplant, it may overlook species-specific interactions and include protein associations that are not present in vivo. These predicted interactions therefore require further validation using approaches such as yeast two-hybrid assays, co-immunoprecipitation, and bimolecular fluorescence complementation.

### Tissue-specific expression profiling of *PP2C* genes in tobacco and eggplant

**Fig. 3:**
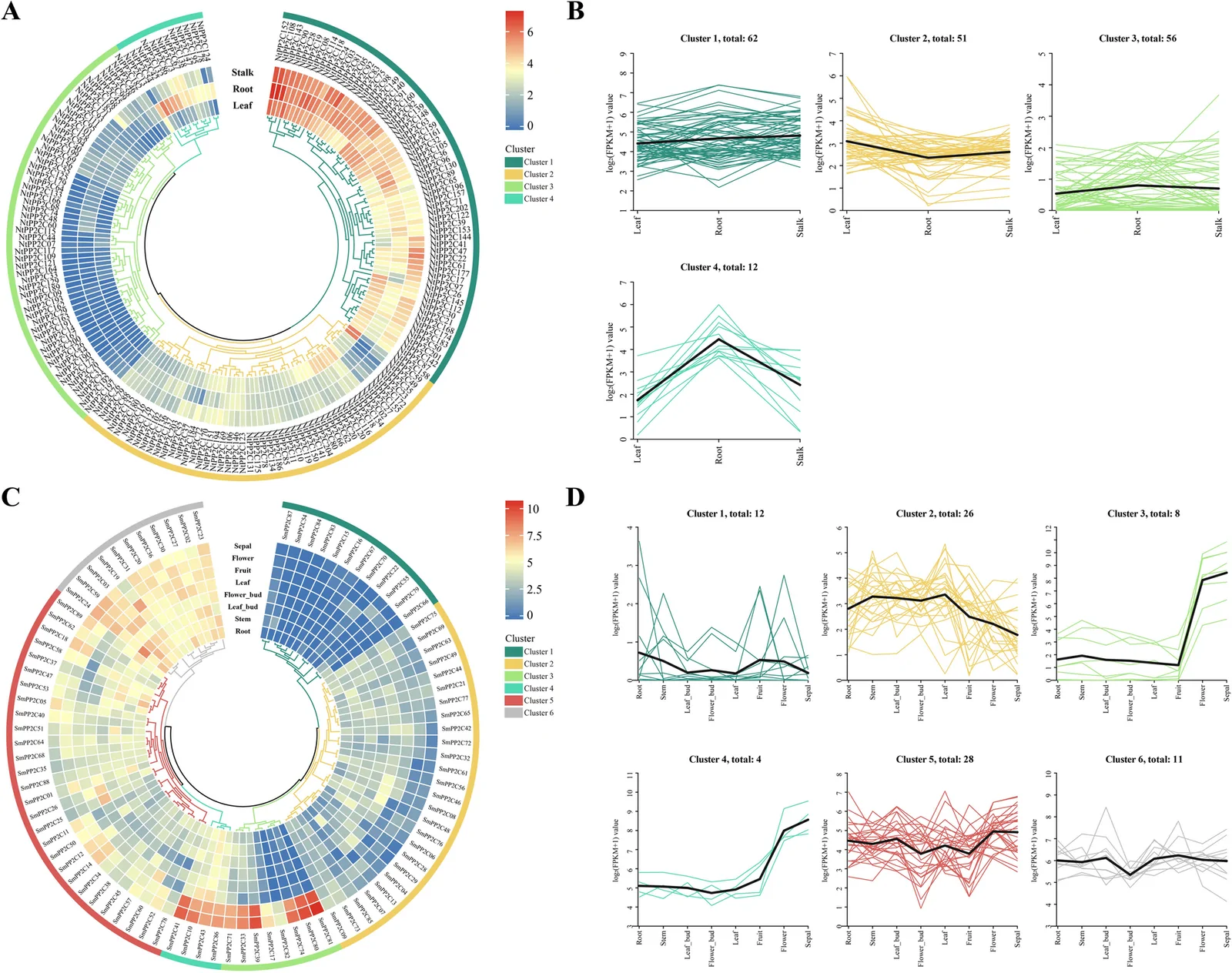
The expression patterns of *NtPP2Cs* and *SmPP2Cs* in different tissues. **A** The expression patterns of *NtPP2Cs.* **B** Line chart of *NtPP2Cs* clustering analysis. **C** The expression patterns of *SmPP2Cs.* **D** Line chart of *SmPP2Cs* clustering analysis. FPKM values for *PP2C* genes were transformed by log_2_(*n* + 1)

Based on RNA-seq data obtained from the NCBI database, the expression profiles of *NtPP2C* and *SmPP2C* genes across various tissues were analyzed to investigate their potential biological functions in tobacco and eggplant. In tobacco, *NtPP2C* genes were classified into four distinct clusters according to their expression patterns (Fig. 3A, B). Genes in cluster 1 exhibited high expression levels in stems, roots, and leaves. In contrast, the genes in cluster 2 showed relatively higher expression in stems and leaves than in roots. The genes in cluster 4 were specifically highly expressed in roots, while those in cluster 3 displayed negligible expression across all the examined tissues. In eggplant, *SmPP2C* genes were categorized into six clusters (Fig. 3C, D). Genes in cluster 1 exhibited minimal expression in all tissues. In contrast, genes in cluster 2 showed low expression in fruits, flowers, and sepals, but had moderate expression in other tissues. Notably, genes in clusters 3 and 4 exhibited significantly elevated expression levels in flowers and sepals. Moreover, the genes in cluster 4 genes had a higher average expression level. Genes in cluster 5 maintained a moderate expression level across tissues, whereas cluster 6 genes exhibited high expression levels in all examined tissues. These findings suggest that genes with tissue-specific high expression may play critical roles in plant growth and development.

### Expression dynamics and regulatory patterns of *PP2C* genes in tobacco and eggplant under abiotic stress

**Fig. 4:**
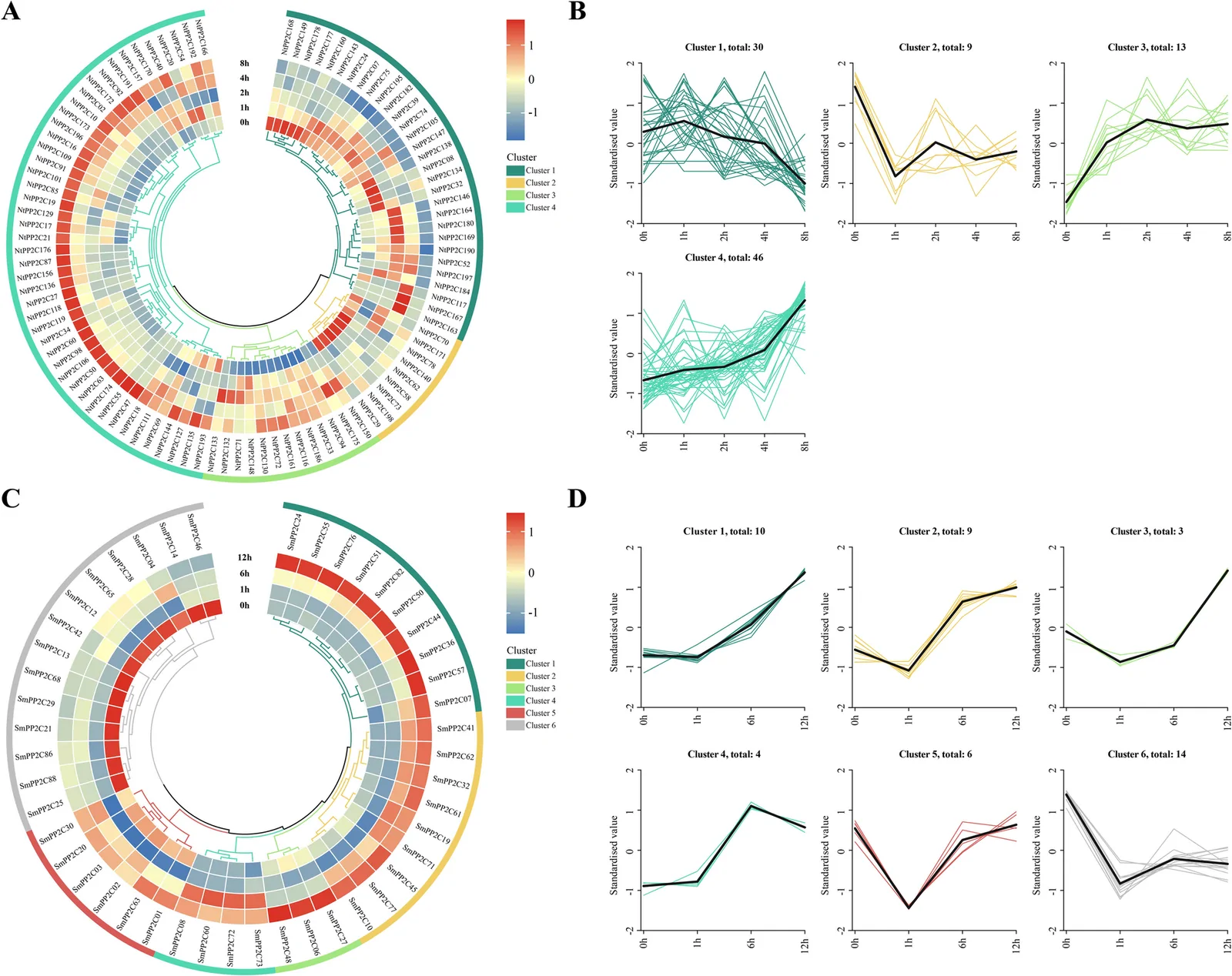
The expression patterns of *NtPP2Cs* and *SmPP2Cs* under abiotic stresses. **A** The expression patterns of *NtPP2Cs* under drought stress. **B** Line chart of *NtPP2Cs* clustering analysis. **C** The expression patterns of *SmPP2Cs* under high temperature. **D** Line chart of *SmPP2Cs* clustering analysis. FPKM values for *PP2C* genes were transformed by log_2_(*n* + 1)

To explore the functions of the *NtPP2C* and *SmPP2C* genes under abiotic stress, the expression levels of *NtPP2Cs* under drought stress and *SmPP2Cs* under heat stress were analyzed using RNA-seq data. The results indicated that the members of the *NtPP2C* and *SmPP2C* gene families exhibited distinct expression patterns under different stress conditions (Fig. 4). In tobacco, the *NtPP2C* genes were classified into four clusters (Fig. 4A, B). The expression of *NtPP2C* genes in cluster 1 gradually increased in response to drought stress, reached a peak and then declined. In clusters 2 and 3, the expression levels of *NtPP2C* genes were down-regulated and up-regulated, respectively, subsequent to drought treatment, followed by fluctuations within a relatively stable range. The expression levels of genes in cluster 4 continuously increased with the prolongation of drought treatment. In eggplant, the *SmPP2C* genes were divided into six clusters (Fig. 4C, D). The expression patterns of *SmPP2C* genes in clusters 1, 2, 3, and 5 were similar, with expression levels decreasing after 1 h of heat treatment, followed by an increase at 6 and 12 h. In cluster 4, gene expression levels steadily increased with the duration of heat treatment, peaking at 6 h before decreasing. In cluster 6, the expression level of *SmPP2C* genes decreased to the lowest point after 1 h of heat treatment, increased at 6 h, and slightly decreased at 12 h. These findings suggest that the *NtPP2C* and *SmPP2C* genes may be involved in the regulation or response of plants to water stress and temperature stress.

### Subcellular localization of NtPP2C91 and SmPP2C24

**Fig. 5:**
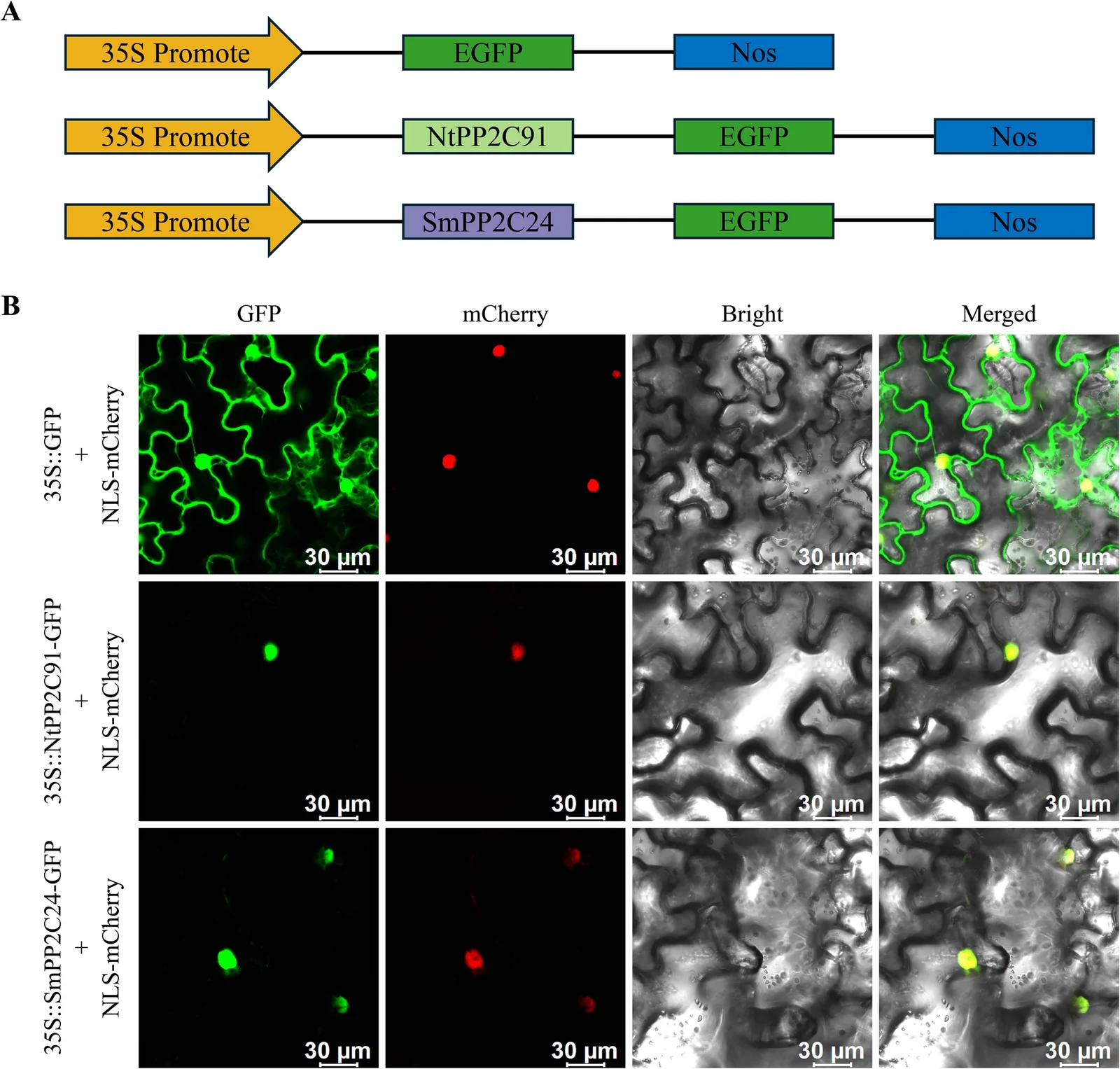
Subcellular localization of *NtPP2C91* and *SmPP2C24*. **A** Construction of *NtPP2C91* and *SmPP2C24* vectors. **B** 35 S::*NtPP2C91*-GFP, 35 S::*SmPP2C24*-GFP and 35 S::GFP (control) were transiently expressed in K326 epidermal cells by *Agrobacterium*-mediated transformation infiltration. Results were observed using confocal microscopy 36 h after transformation. Scale bars = 30 μm

Subcellular localization prediction suggested that the majority of PP2C proteins are localized to the nucleus. To experimentally validate these predictions, *NtPP2C91* and *SmPP2C24* were selected for subcellular localization analysis. We transiently expressed NtPP2C91-GFP and SmPP2C24-GFP fusion proteins in tobacco epidermal cells (Fig. 5A). Fluorescence microscopy confirmed that both proteins are specifically localized to the nucleus (Fig. 5B), consistent with in silico predictions (Table S2).

### Collinearity and phylogenetic analysis of *PP2C* genes in Solanaceae

**Fig. 6:**
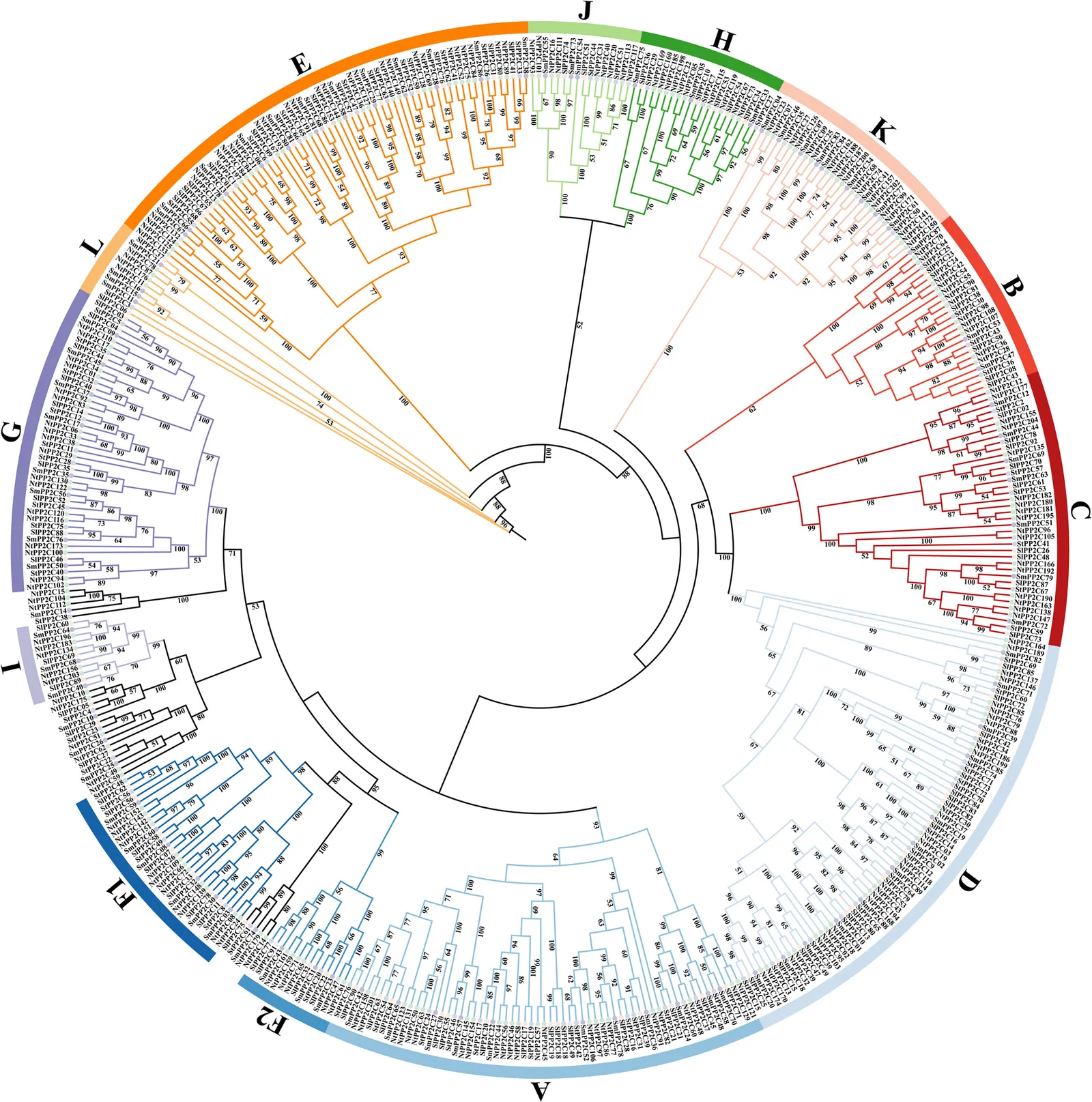
Phylogenetic analysis of NtPP2C from tobacco, SmPP2C from eggplant, and other identified PP2C proteins from various species. Different colored circles represent PP2C proteins from different species

To elucidate the evolutionary relationships among Solanaceae PP2C genes, a phylogenetic tree was constructed using 463 *PP2C* protein sequences from tobacco (204), eggplant (89), tomato (92), and potato (78) (Fig. 6, Table S7). The tree revealed several conserved clades, each containing proteins from all four species, with a notable tendency for tomato and potato proteins to cluster together. In tobacco, NtPP2C proteins were assigned to 12 subgroups (A–L). Subgroups D and E were the largest (28 members each), while subgroup L was the smallest (4 members); 11 NtPP2Cs (NtPP2C10/14/15/49/51/59/62/104/112/175/179) remained unclassified. A similar pattern was observed in eggplant, where subgroups D and E were also the largest (12 members each) and five SmPP2Cs (SmPP2C10/14/25/26/74) were unclassified, and subgroups B, F2, H, I, and J were the smallest, with three proteins each. The consistent subgroup composition across species supports a shared evolutionary origin for the PP2C family in Solanaceae.

**Fig. 7:**
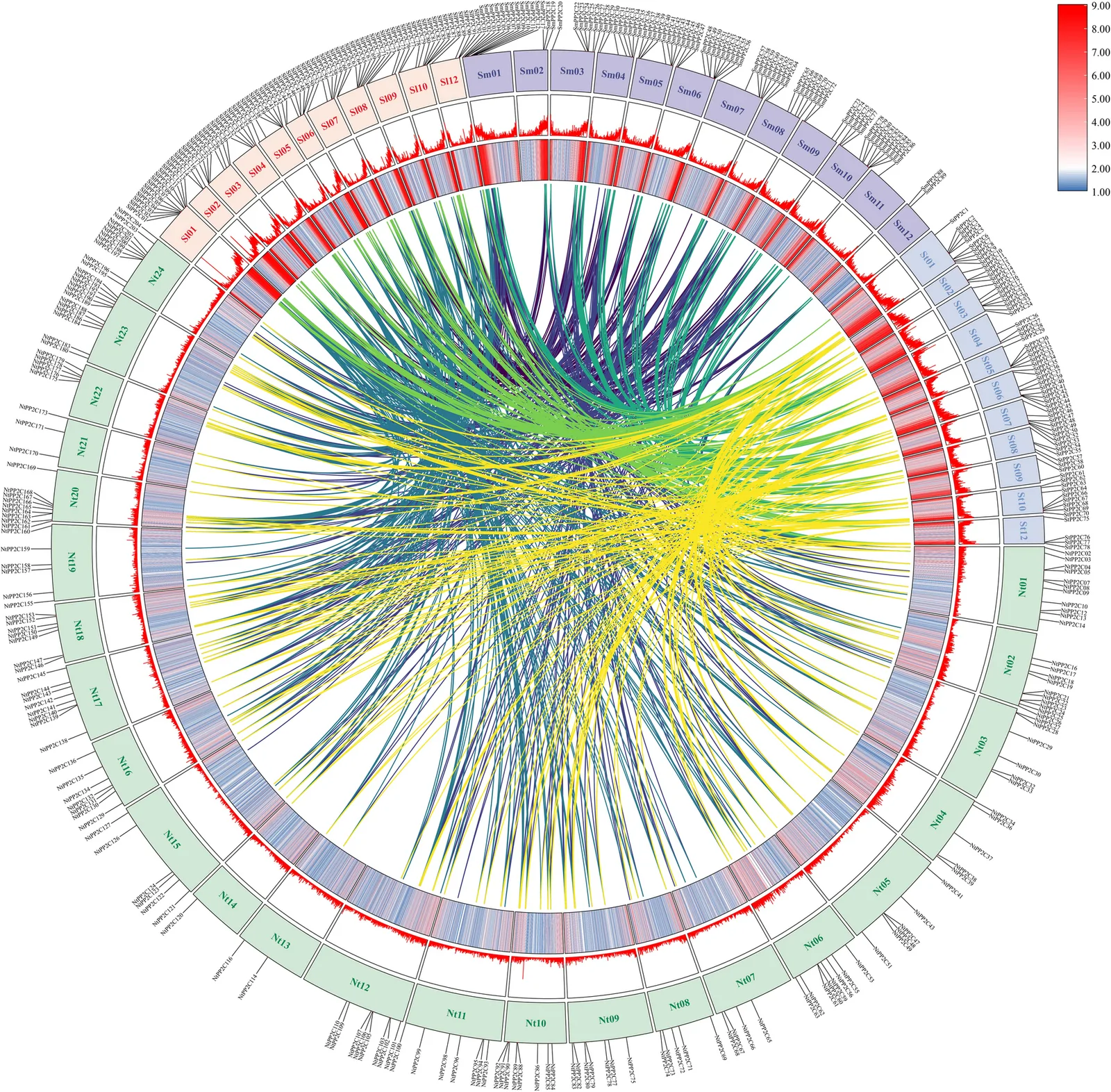
A comparative analysis of the synteny of *PP2C* genes among tobacco, eggplant, tomato, and potato. Different colored lines represent syntenic gene pairs between two species, and different colored rectangles represent chromosomes of different species

Interspecies synteny analysis across the Solanaceae family identified 386 conserved *PP2C* genes and 944 orthologous gene pairs (Fig. 7, Table S8). Among these, tobacco exhibited the highest numbers of orthologous pairs with eggplant (225 pairs) and tomato (215 pairs), indicating a high degree of genomic structure conservation within the family. Furthermore, selective pressure analysis showed that almost all orthologous gene pairs (with one exception) underwent purifying selection (*Ka/Ks* < 1), suggesting that these genes have maintained essential functions throughout evolution (Table S4). Based on previous studies [33, 34], the divergence times of these orthologous gene pairs were further estimated (Table S4). The results showed that tobacco diverged earlier from other Solanaceae species. The larger number of orthologous gene pairs detected between tobacco and other species is likely due to the allotetraploid nature of the tobacco genome.

Subgroup A PP2Cs represent the most extensively characterized subgroup of the PP2C family. Previous studies indicated that they act as central negative regulators in the ABA signaling pathway and play pivotal roles in plant responses to both abiotic and biotic stresses [25]. In *Arabidopsis thaliana*, suppressing the activities of the subgroup A PP2C members, such as *ABI1*, *HAB1*, and *HAI1* enhances tolerance to both drought and salinity [64, 65]. Moreover, *ABI1* can enhance resistance to *Leptosphaeria maculans* by modulating the expression of the *Resistance to Leptosphaeria maculans 1* gene [24]. Notably, *NtPP2C91* and *SmPP2C24* are also classified as subgroup A PP2Cs (Fig. 6) and exhibit high expression levels along with broad tissue distribution (Fig. 3), suggesting that they may play important roles in plant stress responses. Consequently, they were selected for further functional validation.

### *NtPP2C91* knockout enhances cold tolerance and pathogen resistance by regulating the transcriptional dynamics of ABA signaling and PP2C family members

**Fig. 8:**
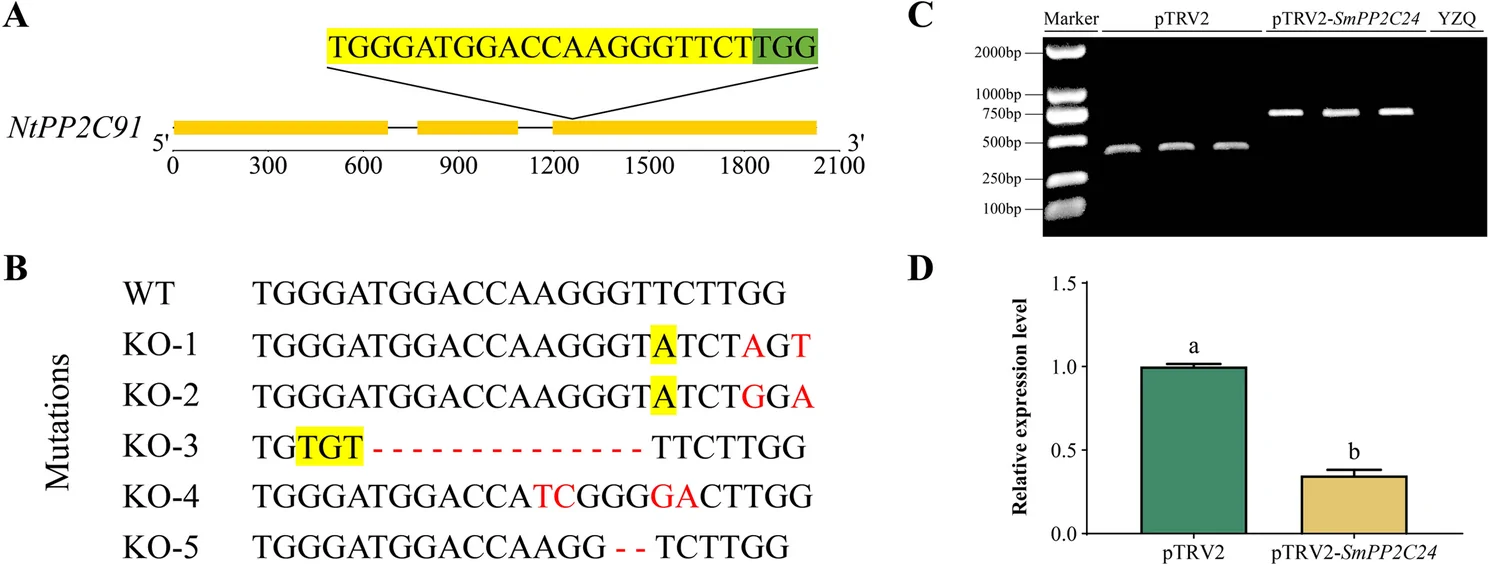
Identification of transgenic plants. **A** Gene structure of *NtPP2C91* and the positions of CRISPR/Cas9 knockout target sites; **B** Mutation types identified in *NtPP2C91* knockout lines; **C** Gel electrophoresis analysis of silenced plants; **D** Relative expression level of *SmPP2C24* in eggplant. Different letters between samples denote significant differences according to one-way ANOVA and Tukey’s test (*P* < 0.05). Error bars are standard deviations of three biological replicates. The eggplant *SmACTIN* gene was used as the reference gene

To investigate the function of *NtPP2C91*, CRISPR/Cas9-mediated knockout targets were designed based on the gene structure. The target site was located in the third exon of *NtPP2C91* (Fig. 8A). Sequencing analysis showed that mutations occurred in five independent plants (Fig. 8B). Among these, a deletion mutant line, KO-5 (designated *ntpp2c91*), was selected, and the T1 generation of this line was used for subsequent stress-treatment experiments.

**Fig. 9:**
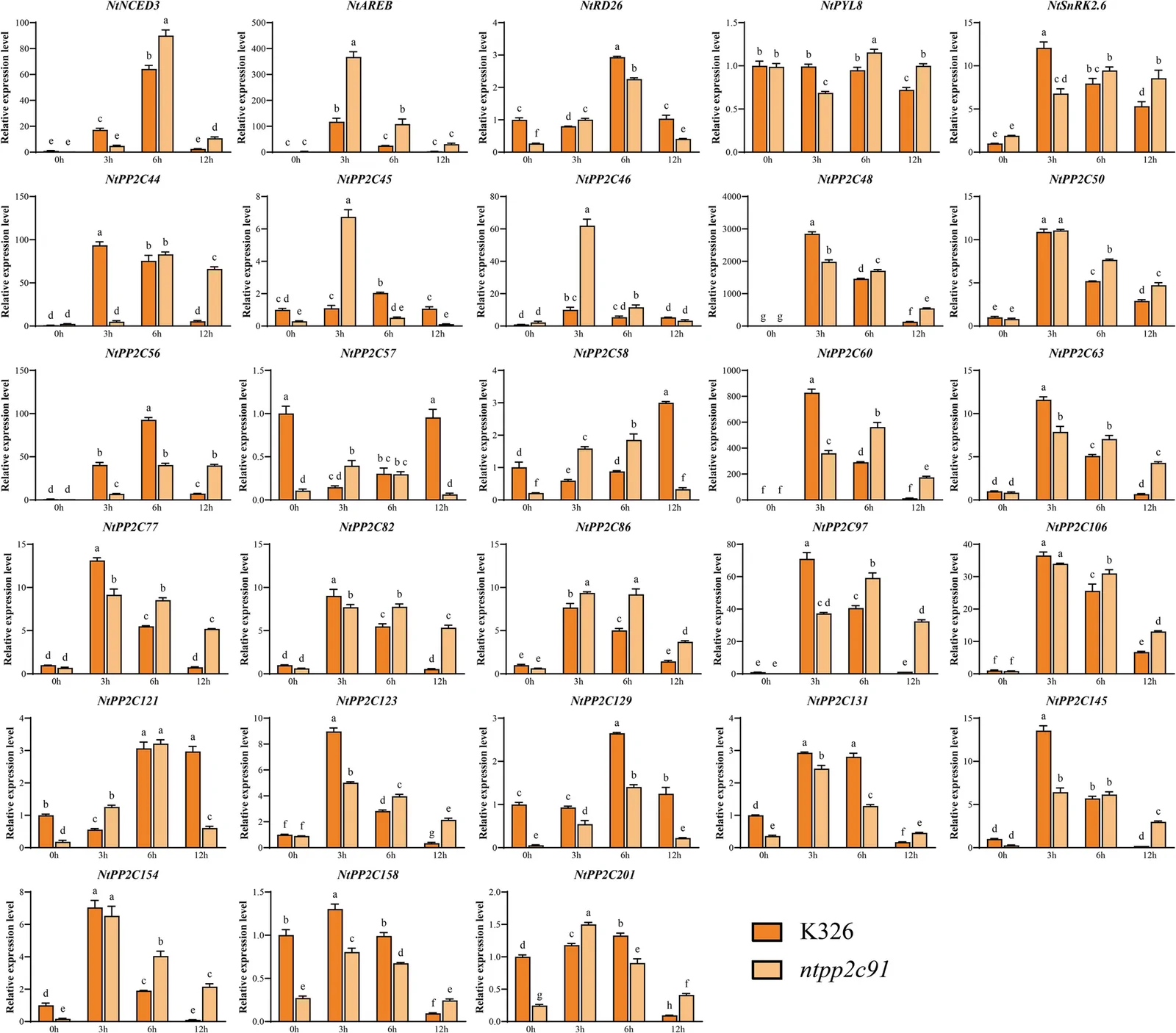
Expression analysis of ABA-responsive genes and *NtPP2C* family genes in leaves of *NtPP2C91*-knockout and control plants under ABA stress. Different letters indicate statistically significant differences at *P* < 0.05 according to two-way ANOVA followed by Tukey’s test for each sample. Error bars are standard deviations of three biological replicates. The tobacco *NtACTIN* gene was used as the reference gene

To elucidate the regulatory role of *NtPP2C91* under ABA stress, homologs within the same subgroup of *NtPP2C91* and several genes associated with the ABA signaling pathway were selected for qRT-PCR analysis following ABA treatment. The results showed that all tested genes responded significantly to ABA, and that knockout of *NtPP2C91* markedly altered their expression patterns (Fig. 9). The key ABA biosynthetic gene *NtNCED3* and ABA signaling-related genes (*NtAREB*, *NtRD26*, *NtPYL8*, and *NtSnRK2.6*) displayed broadly consistent temporal trends in both control plants and knockout plants; however, their transcript levels were generally higher in *ntpp2c91*. Specifically, *NtNCED3*, *NtAREB*, *NtRD26*, and *NtSnRK2.6* exhibited an expression pattern characterized by initial induction followed by a decline, whereas *NtPYL8* showed relatively modest fluctuations. These data indicate that loss of *NtPP2C91* is associated with enhanced activation of ABA biosynthesis and selected regulatory components (*NtNCED3*, *NtAREB*, *NtPYL8*) and with an altered transcriptional response of the core kinase component *NtSnRK2.6*. Moreover, most homologous genes reached their expression peaks at the early (3 h) or mid (6 h) stage after ABA treatment, and their expression decreased by 12 h. Compared with K326, multiple genes (*NtPP2C44/48/50/56/60/63/77/82/86/97/106/123/145*) maintained higher expression levels in *ntpp2c91* during the mid-to-late stage of stress (6–12 h). In addition, *NtPP2C45/46/57/58/86/121/201* were more strongly induced at the early stage in *ntpp2c91*, whereas another set of genes (*NtPP2C44/48/56/60/63/77/82/97/123/129/131/145/158*) showed higher induction levels in K326. Collectively, these results suggest that *NtPP2C91* knockout may influence the overall dynamics of ABA signaling negative feedback by modulating both the magnitude and duration of induction among PP2C family members.

**Fig. 10:**
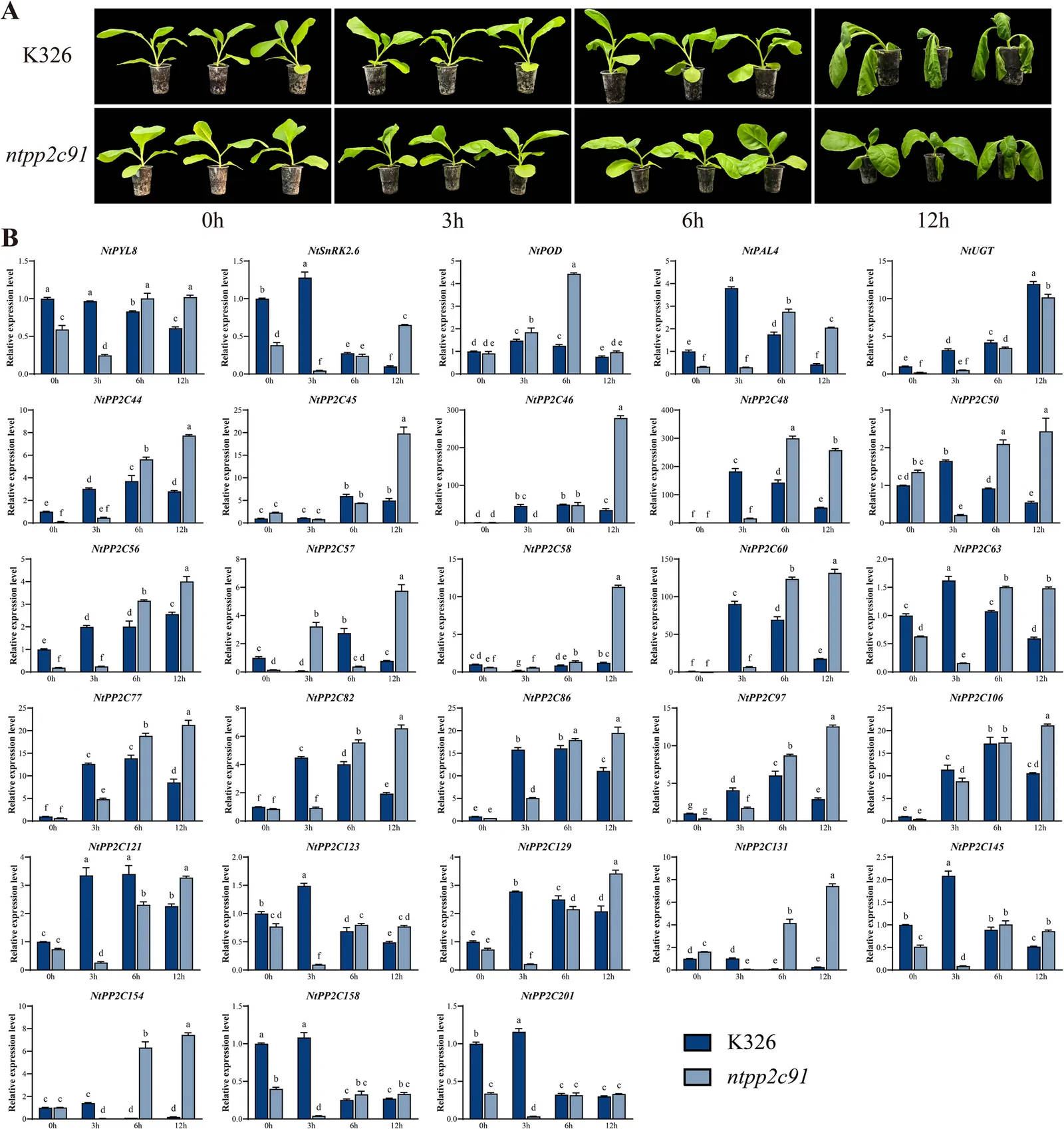
Effects of *NtPP2C91* knockout on the cold-stress response in tobacco. **A** Phenotypic characteristics of *NtPP2C91*-knockout and control plants after cold-stress treatment. **B** Expression analysis of cold-responsive genes and *NtPP2C* family genes in leaves of different tobacco genotypes. Different letters indicate statistically significant differences at *P* < 0.05 according to two-way ANOVA followed by Tukey’s test for each sample. Error bars are standard deviations of three biological replicates. The tobacco *NtACTIN* gene was used as the reference gene

Low-temperature treatment experiments revealed that no obvious phenotypic differences were observed among the plant lines at the early stage (3 h). After 6 h, K326 plants began to display slight leaf wilting. At 12 h after treatment, K326 showed severe wilting, whereas *ntpp2c91* exhibited only mild wilting (Fig. 10A). This phenotypic divergence is associated with the loss of *NtPP2C91* and suggests that *NtPP2C91* negatively influences cold tolerance. Flavonoid biosynthesis is closely associated with antioxidant capacity and stress tolerance in plants [66–72]. QRT-PCR analysis confirmed that all selected genes responded markedly to low-temperature treatment (Fig. 10B). In *ntpp2c91*, the early transcriptional induction of ABA signaling-related genes (*NtPYL8* and *NtSnRK2.6*) and flavonoid biosynthesis–related genes (*NtPAL4* and *NtUGT*) was attenuated, followed by a compensatory upregulation at the mid-to-late stages. Notably, the reactive oxygen species (ROS)-scavenging gene *NtPOD* remained expressed at higher levels in *ntpp2c91* than in K326 throughout the entire treatment period. Collectively, these results are consistent with an enhanced protective response to cold in the absence of *NtPP2C91*, particularly in pathways linked to oxidative stress mitigation. In addition, although the cold-responsive transcriptional program in *ntpp2c91* appeared delayed, the expression levels of *NtPP2C91* homologs (*NtPP2C44/45/46/48/50/56/57/58/60/63/77/82/86/97/106/121/123/129/131/145/154*) were higher than those in the control at later time points, suggesting feedback compensation and potential functional redundancy within the PP2C family.

**Fig. 11:**
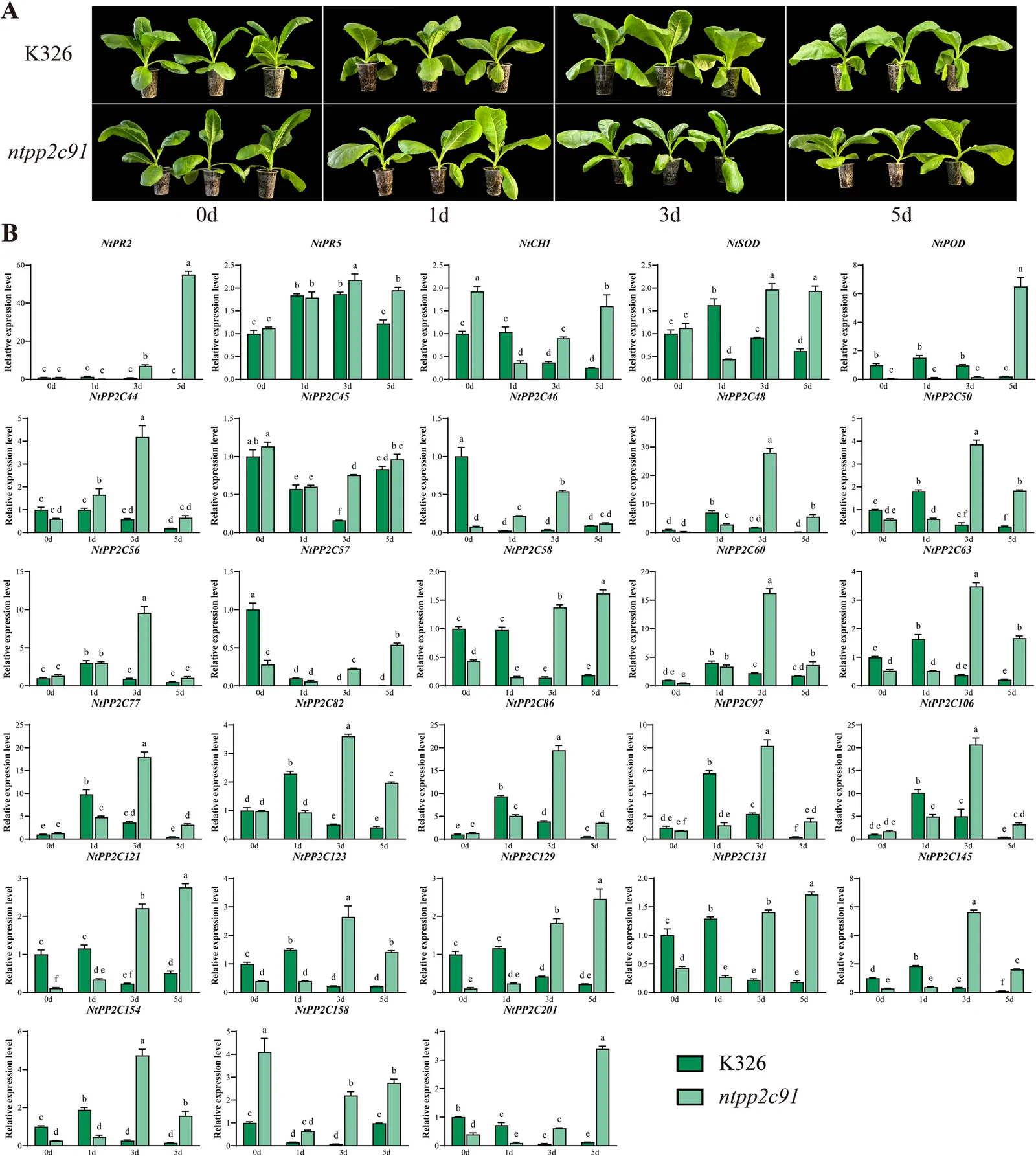
Effects of *NtPP2C91* knockout on tobacco responses to inoculation of *Ras*. **A** Phenotypic characteristics of *NtPP2C91*-knockout and control plants after *Ras* inoculation. **B** Expression analysis of defense-related genes and *NtPP2C* family genes in leaves of different tobacco genotypes. Different letters indicate statistically significant differences at *P* < 0.05 according to two-way ANOVA followed by Tukey’s test for each sample. Error bars are standard deviations of three biological replicates. The tobacco *NtACTIN* gene was used as the reference gene

In the *Ras* infection assay, K326 plants exhibited slight leaf wilting at 1 day post-inoculation (dpi), whereas *ntpp2c91* plants showed no apparent phenotypic changes. By 3 dpi, wilting in K326 became more pronounced, while *ntpp2c91* plants began to display mild wilting. At 5 dpi, wilting symptoms in K326 progressed upward from the lower leaves and were markedly more severe than those in *ntpp2c91* (Fig. 11A). Collectively, these observations suggest that *NtPP2C91* may act as a negative regulator of tobacco responses to *Ras* infection, and that loss of *NtPP2C91* function enhances plant resistance to the pathogen. Consistent with this phenotypic difference, qRT–PCR analysis showed that several defense-related genes (*NtPR2*, *NtPR5*, *NtCHI*, *NtSOD*, and *NtPOD*) were more strongly transcriptionally activated in *ntpp2c91* than in the control during the mid-to-late stages of infection (3–5 dpi) (Fig. 11B). In addition, expression of *NtPP2C91* homologous genes (*NtPP2C48/50/58/63/77/82/86/97/106/121/123/129/131/145/154/201*) in *ntpp2c91* was generally lower than in the control at 1 dpi but became significantly higher at 3–5 dpi. This pattern indicates a shift in the responsiveness of these homologs from an early, transient induction to a stronger induction at later stages, which may reflect compensatory regulation following *NtPP2C91* disruption.

### Silencing *SmPP2C24* enhances cold tolerance and pathogen resistance by regulating the transcriptional dynamics of ABA signaling and PP2C family members in eggplant

To investigate the potential role of *SmPP2C24* in hormone signaling, cold tolerance, and pathogen stress responses in eggplant, *SmPP2C24* was silenced using VIGS technology. PCR amplification performed 14 days after agroinfiltration detected a fragment of the expected size, confirming the successful introduction of the recombinant vector into inoculated plants (Fig. 8C). Silencing efficiency was then assessed by qRT-PCR. Compared with the control, *SmPP2C24* transcript levels were significantly reduced in the silenced plants (Fig. 8D), indicating that *SmPP2C24* silencing was successful.

**Fig. 12:**
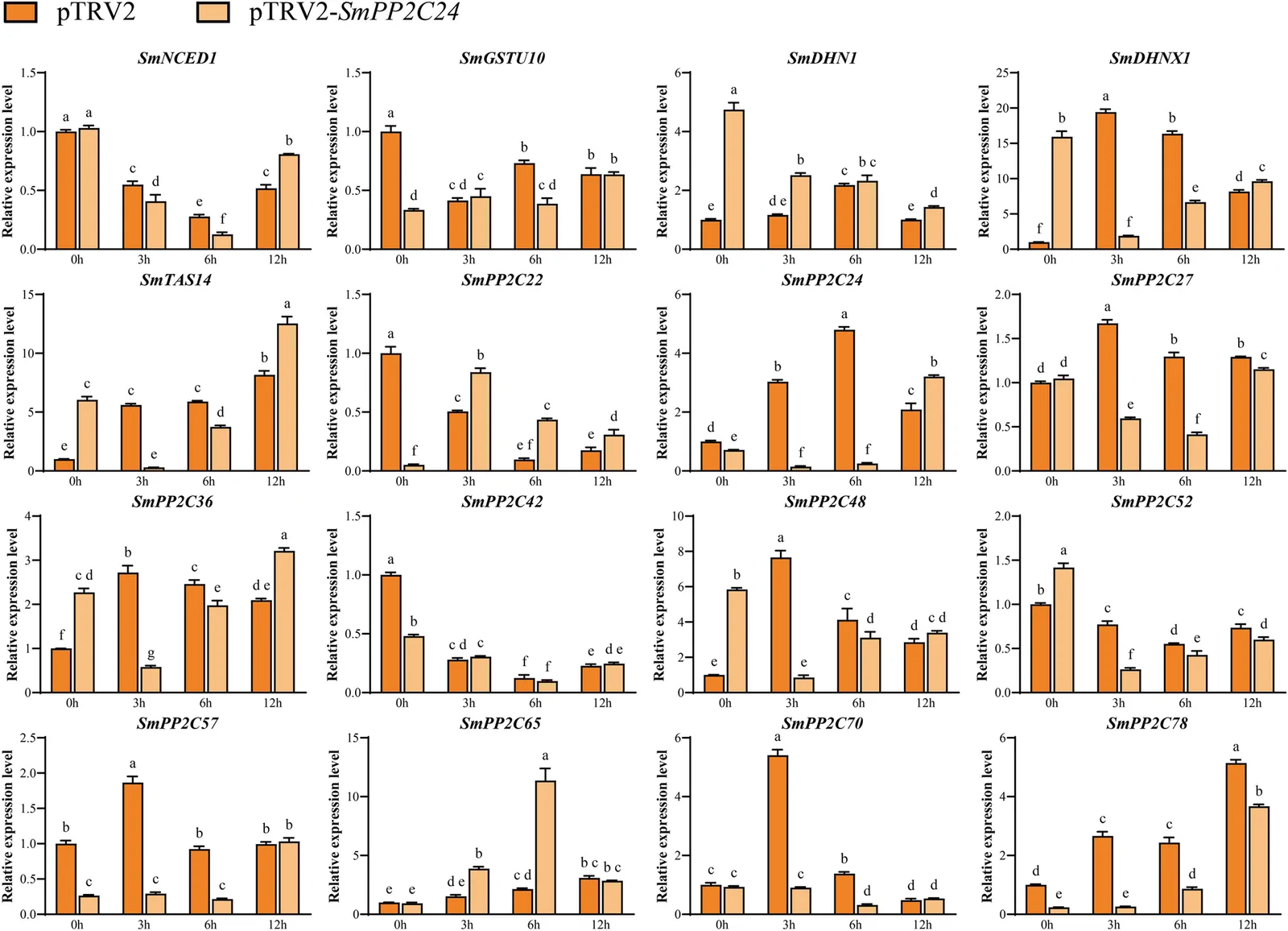
Expression analysis of ABA-responsive genes and *SmPP2C* family genes in leaves of *SmPP2C24*-VIGS and control plants under ABA stress. Different letters indicate statistically significant differences at *P* < 0.05 according to two-way ANOVA followed by Tukey’s test for each sample. Error bars are standard deviations of three biological replicates. The eggplant *SmACTIN* gene was used as the reference gene

Plants from the control pTRV2 line and the *SmPP2C24*-silenced pTRV2-SmPP2C24 line were treated with ABA, and transcripts were measured at 0, 3, 6, and 12 h for an ABA biosynthesis gene (*SmNCED1*), ABA signaling-related genes (*SmGSTU10*, *SmDHN1*, *SmDHNX1*, and *SmTAS14*), and subgroup A SmPP2Cs (Fig. 12). In pTRV2 plants, *SmPP2C24* was strongly induced by ABA. In pTRV2-*SmPP2C24* plants, *SmPP2C24* transcripts were significantly reduced at 3 and 6 h but rebounded at 12 h to above control levels, indicating that gene silencing was most effective during the early phase of ABA treatment. Under ABA treatment, control plants displayed a typical rapid early transcriptional induction, most prominently for *SmDHNX1* and *SmTAS14*. By comparison, pTRV2-*SmPP2C24* plants exhibited a composite response characterized by elevated basal expression of ABA-responsive genes, attenuated early inducibility, and a compensatory late rebound. These patterns suggest that silencing of *SmPP2C24* shifts the ABA pathway toward a higher basal activation state, thereby weakening the early induction triggered by exogenous ABA. Notably, *SmPP2C24* homologs showed divergent responses in the silenced background. *SmPP2C22* and *SmPP2C65* were significantly upregulated, whereas the early induction of other homologs (*SmPP2C27/57/70/78*) was reduced or delayed. Collectively, these results support a role for *SmPP2C24* in maintaining the dynamic balance of ABA signaling, and its silencing substantially reprograms the temporal pattern of transcriptional responses to ABA.

**Fig. 13:**
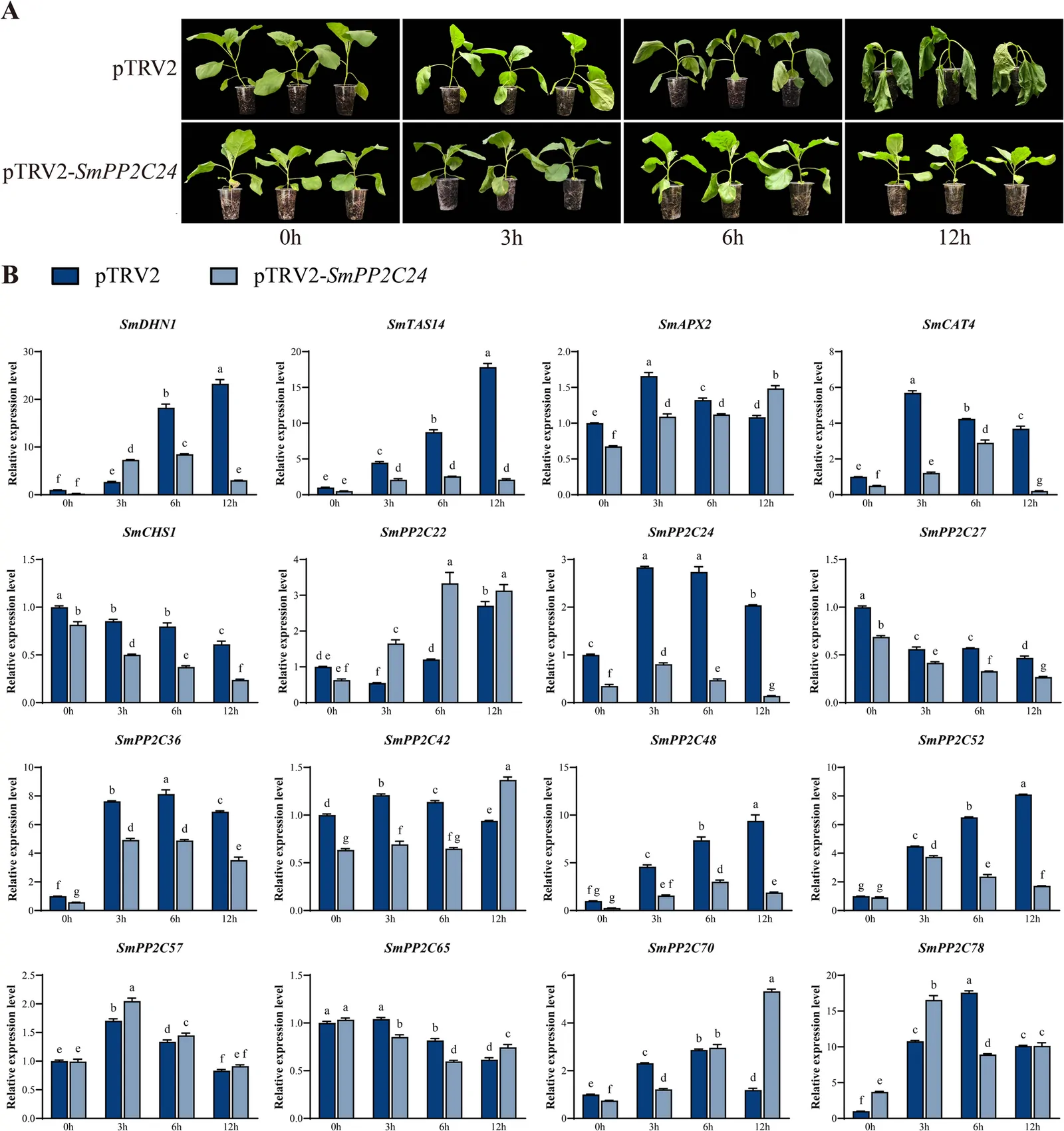
Effects of *SmPP2C24* silencing on the cold-stress response in eggplant. **A** Phenotypic characteristics of *SmPP2C24*-VIGS and control plants after cold-stress treatment. **B** Expression analysis of cold-responsive genes and *SmPP2C* family genes in leaves of different eggplant genotypes. Different letters indicate statistically significant differences at *P* < 0.05 according to two-way ANOVA followed by Tukey’s test for each sample. Error bars are standard deviations of three biological replicates. The eggplant *SmACTIN* gene was used as the reference gene

Low-temperature treatment experiments revealed that both the control plants and the silenced plants exhibited vigorous growth at 0 h. After 3 h of treatment, the pTRV2 plants began to show slight leaf wilting. By 6 h, the phenotypic differences became more pronounced, with the pTRV2 plants displaying obvious wilting. After 12 h, the pTRV2 plants exhibited severe wilting, whereas the pTRV2-*SmPP2C24* plants largely maintained normal growth (Fig. 13A). These observations suggest that silencing *SmPP2C24* may enhance cold tolerance in eggplant. qRT-PCR analysis confirmed a significant reduction in *SmPP2C24* transcript abundance in pTRV2-SmPP2C24 plants relative to pTRV2 controls, indicating efficient gene silencing (Fig. 13B). Moreover, the cold-induced expression of ABA signaling-related genes (*SmDHN1* and *SmTAS14*), ROS-scavenging genes (*SmAPX2* and *SmCAT4*), and a flavonoid biosynthetic gene (*SmCHS1*) was greatly reduced in the *SmPP2C24*-silenced background. Notably, *SmPP2C22*, *SmPP2C57* and *SmPP2C78* were upregulated in pTRV2-*SmPP2C24* plants, whereas the expression of other subgroup A *SmPP2C* genes (*SmPP2C27/36/42/48/52/65/70*) was significantly decreased, suggesting that *SmPP2C24* silencing substantially alters the transcriptional responses of homologous *PP2C* genes during cold stress.

**Fig. 14:**
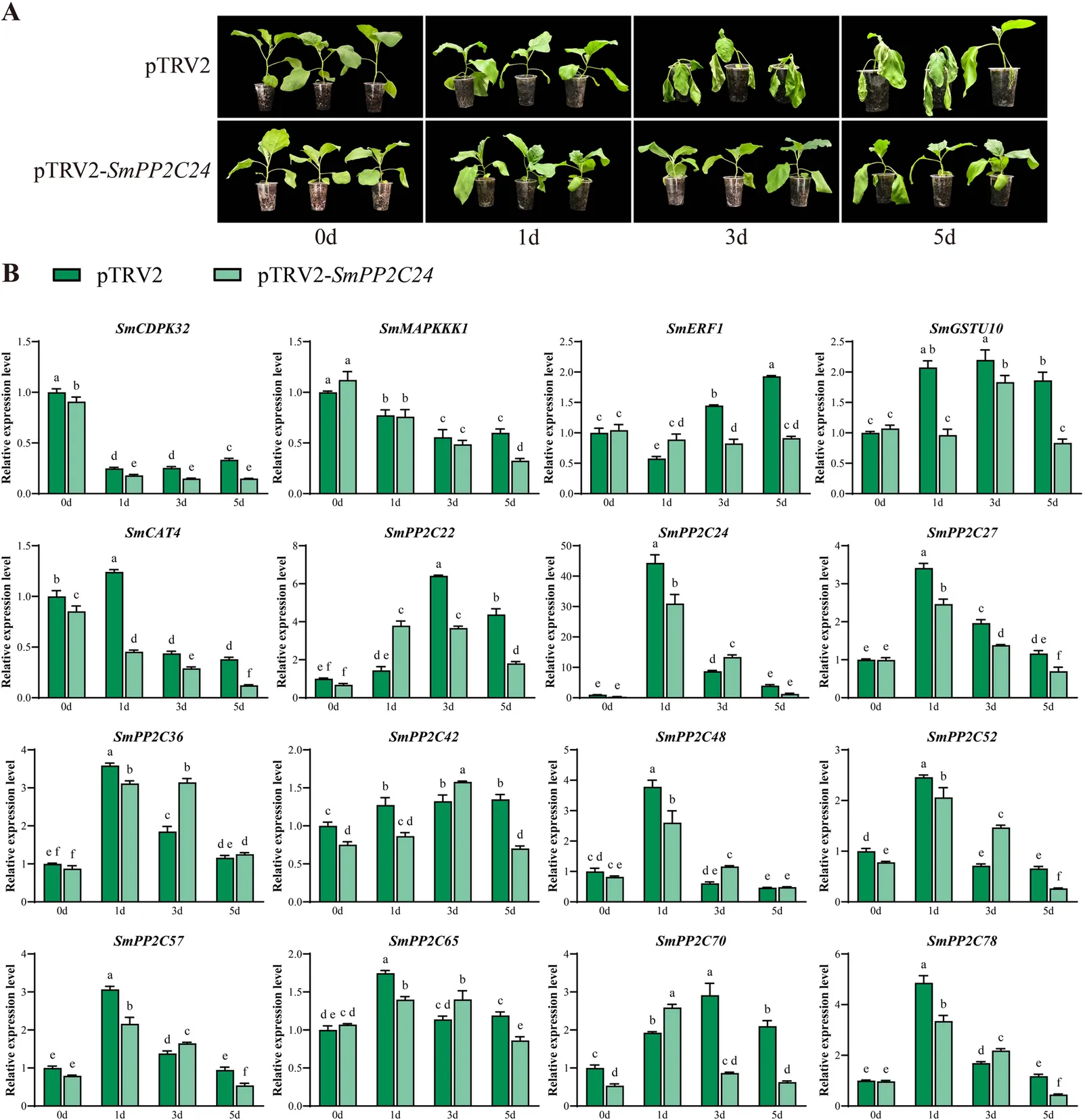
Effects of *SmPP2C24* silencing on eggplant responses to inoculation of *Ras*. **A** Phenotypic characteristics of *SmPP2C24*-VIGS and control plants after *Ras* inoculation. **B** Expression analysis of defense-related genes and *SmPP2C* family genes in leaves of different eggplant genotypes. Different letters indicate statistically significant differences at *P* < 0.05 according to two-way ANOVA followed by Tukey’s test for each sample. Error bars are standard deviations of three biological replicates. The eggplant *SmACTIN* gene was used as the reference gene

In the *Ras* infection assay, no obvious symptoms were observed in either pTRV2 or pTRV2-*SmPP2C24* plants at 1 dpi. At 3 dpi, pTRV2 plants exhibited severe wilting, whereas pTRV2-*SmPP2C24* plants remained largely phenotypically normal. By 5 dpi, the phenotype of pTRV2 plants showed little further change, while pTRV2-*SmPP2C24* plants developed only mild wilting symptoms (Fig. 14A). These results indicate that silencing *SmPP2C24* markedly enhances plant resistance to *Ras*. To further investigate the role of *SmPP2C24* in the infection response, we analyzed the transcript levels of disease resistance-related genes (*SmCDPK32*, *SmMAPKKK1*, *SmERF1*, *SmGSTU10*, and *SmCAT4*) as well as subgroup A *SmPP2C* genes (*SmPP2C22/24/27/36/42/48/52/57/65/70/78*) following *Ras* infection by qRT-PCR (Fig. 14B). Compared with the control plants, the selected genes were generally downregulated in pTRV2-SmPP2C24 plants. Combined with the phenotypic data, this result suggests that the attenuated transcriptional induction observed in pTRV2-*SmPP2C24* plants may be associated with reduced infection intensity or altered defense signaling dynamics. Together, these findings indicate that *SmPP2C24* is involved in the plant response to *Ras* infection and influences the expression dynamics of related genes.

## Discussion

The *PP2C* gene family plays pivotal roles in a wide range of physiological processes involved in plant growth and development [73]. In this study, we integrated genome-wide identification, evolutionary analysis, stress-responsive expression profiling, and targeted functional validation to establish a comparative genomic and functional framework for PP2C *genes* in Solanaceae species. Through genome-wide analysis, we identified 204 *NtPP2C* genes in tobacco and 89 *SmPP2C* genes in eggplant. The number of PP2C genes in tobacco was substantially higher than those reported in Arabidopsis (80) and rice (78), and was more comparable to the expanded PP2C repertoire observed in polyploid wheat [16, 19]. This feature is consistent with the allotetraploid origin of tobacco and further supports the general notion that gene duplication is a major driving force underlying the expansion of regulatory gene families in plants [74, 75]. In the present study, segmental duplication represented the predominant duplication mode, suggesting that large-scale genomic duplication events contributed substantially to the expansion of the PP2C gene family. Comparative analyses revealed that the PP2C family is highly conserved during evolution, while also exhibiting substantial structural diversity. This coexistence of evolutionary conservation and structural diversification suggests that PP2C family members have retained core regulatory functions while undergoing functional divergence during species evolution. More importantly, using CRISPR/Cas9-mediated knockout and VIGS, this study revealed that the subgroup A PP2C members *NtPP2C91* and *SmPP2C24* are involved in regulating plant tolerance to low temperature and immune responses against bacterial wilt pathogen infection, where they appear to act as negative regulators. These genes may modulate plant stress adaptation, at least in part, by affecting the transcriptional responses of hormone signaling-related genes and by participating in regulatory interactions within the PP2C family. The findings can guide multiplex gene edits to optimize trade-offs between growth and stress responses, ultimately contributing to the development of climate-resilient crops with enhanced field performance under fluctuating environmental conditions.

Structural and evolutionary analyses of the PP2C family in Solanaceae suggest a substantial degree of functional conservation. Both *NtPP2C* and *SmPP2C* genes showed a conserved 3-2-1 motif distribution pattern (Figure S2B), which is closely associated with the catalytic core domain required for serine/threonine dephosphorylation [76, 77]. In addition, most homologous gene pairs showed *Ka/Ks* < 1, indicating that these genes have been subjected to purifying selection and strong evolutionary constraints, likely to maintain the integrity of the catalytic core domain. In contrast to the conserved protein features, the promoter regions of these genes contained diverse *cis*-acting regulatory elements (Figure S3), suggesting potential divergence in transcriptional regulation. This observation is consistent with previous studies showing that PP2C family members are often involved in multiple stress-response pathways rather than being restricted to a single environmental stimulus. In different plant species, PP2Cs have been reported to participate not only in ABA-related drought and osmotic stress signaling, but also in cold responses and plant immunity, indicating that functional overlap within this family is common [24]. For example, subgroup A PP2Cs in maize are induced by multiple abiotic stresses [25], Medicago PP2Cs respond differentially to drought and cold [78], and Arabidopsis PP2C15 negatively regulates pattern-triggered immunity through BAK1 [79]. Consistent with these reports, *NtPP2C91* and *SmPP2C24* were initially selected because they were significantly induced under drought and high-temperature stress, and subsequent functional analyses further indicated that both genes are involved in the regulation of cold tolerance and disease resistance. These results suggest that *NtPP2C91* and *SmPP2C24* may not act exclusively in a single stress-response pathway, but instead may participate in the crosstalk between biotic and abiotic stress signaling in Solanaceae species. Their broad responsiveness to multiple stresses may be partly associated with the enrichment of stress-related cis-acting elements in their promoters, including the low-temperature-responsive element LTR, the biotic/abiotic stress-associated W-box, and the ABA-responsive element ABRE (Figure S3). These elements may serve as potential regulatory nodes for integrating distinct stress signals and coordinating whole-plant adaptive responses.

Functional analysis showed that both *NtPP2C91* knockout and *SmPP2C24* silencing enhanced plant resistance to biotic and abiotic stresses. Although these two genes were examined in different solanaceous species and through different loss-of-function approaches, their similar resistance-associated phenotypes are consistent with the conserved role of PP2C proteins as negative regulators of ABA- and stress-related signaling. Previous studies have indicated that ABA levels in plant cells remain low under normal conditions, and PP2C phosphatases inhibit the activity of sucrose non-fermenting related protein kinase 2 (SnRK2) [80]. In contrast, under stress conditions, ABA is perceived by the pyrabactin resistance (PYR)/pyrabactin resistant-like (PYL)/ABA receptor regulatory component (RCAR) [80]. Therefore, suppression of PP2C function may release ABA-dependent and stress-adaptive responses, including transcriptional reprogramming, stomatal regulation, antioxidant responses, and ion homeostasis [80–82]. Thus, the consistent increase in stress resistance observed in both *ntpp2c91* and pTRV2-*SmPP2C24* plants suggests that suppression of PP2C function may represent a broadly conserved strategy for enhancing stress adaptation, rather than a species- or method-specific phenomenon. Nevertheless, the expression patterns of defense-related genes differed between the two lines. Compared with their respective controls, defense-related genes in *ntpp2c91* plants were strongly induced at the middle and late stages, whereas this induction was weaker in pTRV2-*SmPP2C24* plants. This difference does not necessarily indicate reduced resistance in *SmPP2C24*-silenced plants. Instead, *SmPP2C24* silencing may have altered the basal state of ABA- and stress-related signaling, as reflected by the higher transcript levels at 0 h, thereby placing the plants in a more primed state. Such priming may enable earlier or faster defense activation upon stress exposure while avoiding the high fitness costs associated with constitutive defense activation [83, 84]. Therefore, the relatively weaker late-stage induction of defense-related genes in pTRV2-*SmPP2C24* plants may reflect more efficient early defense activation, reduced stress pressure at later stages, or a shift in defense output toward physiological regulation, such as stomatal control and ion homeostasis, rather than a reduction in resistance itself [80, 83, 84]. It should be noted, however, that while five independent T0 *NtPP2C91*-edited lines were generated, the stress phenotyping was conducted using the T1 progeny of a single deletion line, and the eggplant assays relied on a single VIGS construct targeting *SmPP2C24*. Therefore, our conclusions are necessarily limited to the tested line and construct, and confirmation through independent mutant alleles and complementation experiments is required.

The targeted knockdown and knockout analyses performed in this study suggest that compensatory regulation may occur within the *PP2C* gene family. Specifically, in *NtPP2C91*-knockout tobacco plants and *SmPP2C24*-silenced eggplant plants, impairment of the target genes did not lead to a pronounced disruption of signaling homeostasis. Instead, the expression levels of several homologous *PP2C* genes in subgroup A were altered, indicating that these homologs may participate in a transcriptional compensatory response. Based on these observations, we propose a working model in which the loss or attenuation of target gene function may result in enhanced activation of downstream SnRK2 kinases, thereby increasing the phosphorylation level and transcriptional activity of AREB transcription factors [80]. Activated AREB proteins could subsequently bind to ABA-responsive element-enriched regions in the promoters of paralogous *PP2C* genes, promoting their expression and thereby contributing to the restoration of signaling homeostasis. This hypothesis is consistent with recent evidence suggesting that PP2C family genes are not only functionally redundant but may also act cooperatively in pathway regulation [85]. Taken together, this putative negative-feedback regulatory mechanism may help explain why a large number of expanded *PP2C* genes have been retained during the evolution of solanaceous plants. Such a mechanism could enhance the stability of core developmental and stress-response pathways, allowing plants to maintain regulatory plasticity under genetic perturbation or environmental fluctuation. However, PP2C enzymatic activity, PYL/SnRK2-binding capacity, and target phosphorylation levels were not examined in the present study. Therefore, the current data support an association between phenotype and transcriptional regulation and suggest a potential negative regulatory role, but they are not sufficient to establish the complete biochemical mechanism underlying this process.

Unlike previous PP2C studies that were largely confined to single species or lacked functional validation, this study integrates comparative evolutionary analysis with cross-species functional verification by applying a unified phylogenetic and synteny-based framework across tobacco, eggplant, tomato, and potato. This approach clarifies the conserved features, expansion patterns, and species-specific functional divergence of *PP2C* genes in Solanaceae. Further functional analyses of *NtPP2C91* and *SmPP2C24* demonstrate that subgroup A PP2Cs are not restricted to the canonical ABA signaling pathway, but also participate in the regulation of cold tolerance and bacterial wilt resistance. From a breeding perspective, these two genes represent promising candidate targets for improving stress resilience in solanaceous crops. Strategies such as gene editing, elite allele mining, or fine-tuning of PP2C expression may enhance crop tolerance to low temperature and resistance to bacterial wilt, although the potential trade-offs among stress resistance, plant growth, development, and hormone sensitivity should be systematically evaluated. Overall, this study identifies *NtPP2C91* and *SmPP2C24* as evolutionarily conserved stress-responsive regulators with species-specific regulatory effects, providing a foundation for further elucidating PP2C-mediated signaling networks in Solanaceae.

## Conclusion

**Fig. 15:**
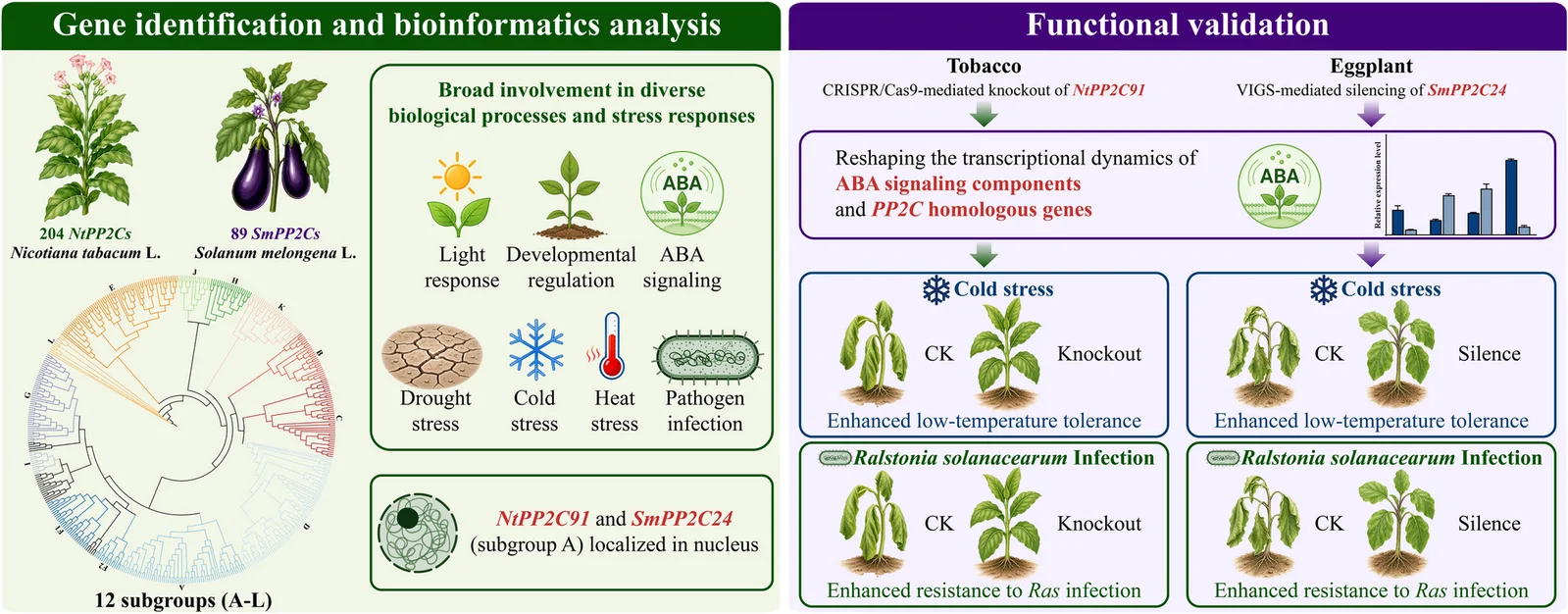
Schematic overview of PP2C gene identification, bioinformatic characterization, and functional validation in two Solanaceae species. A total of 204 *PP2C* genes in tobacco and 89 in eggplant were identified and classified into 12 subgroups. Comprehensive bioinformatic analyses implicated these genes in diverse biological processes and stress-responsive regulatory pathways. Subcellular localization confirmed the nuclear localization of the subgroup A members *NtPP2C91* and *SmPP2C24*. Loss-of-function or silencing of these two genes reshaped the transcriptional profiles of ABA signaling components and PP2C homologs, thereby enhancing plant tolerance to low temperature and resistance to *Ralstonia solanacearum* infection

This study conducted a comprehensive genomic and functional analysis of the *PP2C* gene family in tobacco and eggplant, two economically important crops in the Solanaceae family. A total of 204 *NtPP2C* genes and 89 *SmPP2C* genes were identified and classified into 12 subgroups, designated A to L. Analyses of gene duplication events and selection pressure provided insights into the evolutionary expansion of this gene family. Further analyses of *cis*-acting elements, protein-protein interaction networks, and gene expression profiles indicated that PP2C family members may be involved in plant hormone signaling, as well as responses to abiotic and biotic stresses. Functional validation of two representative subgroup A *PP2C* genes, *NtPP2C91* and *SmPP2C24*, showed that they negatively regulate ABA signaling, cold tolerance, and resistance to bacterial wilt (Fig. 15). Overall, these findings improve our understanding of the functional characteristics of the *PP2C* gene family in Solanaceae plants and provide a theoretical basis for identifying key genes associated with environmental stress resistance in crops.

## Supplementary Information

Supplementary Material 1: Figure S1 Leaves of Wild-type (WT) plants and plants infected with the pTRV2-GFP vector were observed under ultraviolet illumination. Figure S2 Conserved motifs and exon-intron structure analysis of PP2C family in Nicotiana tabacum L. and Solanum melongena L. (A) Phylogenetic tree showing that NtPP2C and SmPP2C genes are clustered into distinct subgroups, with each subgroup indicated by a different color. (B) Conserved motifs of the PP2C gene family, with different colored boxes denoting distinct conserved motifs. (C) Exon–intron structures of NtPP2C and SmPP2C genes. Green boxes represent UTRs, and yellow boxes represent CDSs. Figure S3 Cis-element analysis in the promoters of NtPP2Cs and SmPP2Cs. (A) Different colors represent the different types of cis-elements. (B) The intensity of red represents the number of cis-elements in the promoter of NtPP2Cs and SmPP2Cs.

Supplementary Material 2.

## Data Availability

All data generated or analyzed during this study are included in this published article and its supplementary information files.
